# Duration and barriers of physical education teaching in primary schools in 6 French departments

**DOI:** 10.1371/journal.pone.0362195

**Published:** 2026-11-03

**Authors:** Eric Hermand, Serge Durali, Hugues Rolan, Laurence Munoz, Thierry Pezé, Valentin Smee

**Affiliations:** 1 Université Sorbonne Paris Nord, Hypoxie et Poumon, Bobigny, Seine-Saint-Denis, France; 2 Département STAPS, Université Sorbonne Paris Nord, Bobigny, Seine-Saint-Denis, France; 3 Université Littoral Côte d’Opale, Université Artois, Université Lille, CHU Lille, ULR - URePSSS - Unité de Recherche Pluridisciplinaire Sport Santé Société, Dunkerque, Nord, France; 4 Université Littoral Côte d’Opale, Calais, Pas-de-Calais, France; University of Salerno: Universita degli Studi di Salerno, ITALY

## Abstract

**Purpose:**

Regular physical activity plays an important role in preventing sedentary lifestyles and reducing the risk of chronic diseases. In French primary schools, Physical Education (PE) represents the main compulsory opportunity for structured physical activity during school hours. This study aimed to assess self-reported weekly PE duration among primary school teachers in six French departments and to identify teacher- and school-related factors associated with PE duration, as well as the main obstacles to PE delivery reported by teachers.

**Methods:**

Between March 2021 and June 2024, as the survey implementation in the different areas took place, an anonymous online questionnaire was sent to primary school teachers in six French departments about the time spent teaching PE and their professional habits in this subject.

**Results:**

A total of 1,967 responses (overall response rate 3.9%) were collected and analysed: mean weekly PE duration reached 2 h 23 min ± 1 h 04 min in preschool and 2 h 28 min ± 51 min in elementary school, both below the mandatory programme durations. Higher PE duration was associated with longer seniority, fewer pupils in class, longer peri-activity around PE (transportation, locker room), multigrade class, suburban schools, the implementation of APQ (Activité Physique Quotidienne, a novel compulsory subject focused on daily physical activity), a higher feeling of competence and fewer obstacles declared.

**Conclusions:**

In this sample of primary school teachers from six French departments, self-reported PE duration was below the durations recommended by nationals programmes, consistent with previous French studies. Because of its cross-sectional nature, self-report survey conducted in a limited number of departments, no causal relationships can be inferred and the findings should not be generalized to France as a whole. Nonetheless, this study identifies several teacher- and school-related factors associated with PE duration that may warrant further investigation, including through longitudinal or intervention research. Given the essential role of physical activity in the health of children and future adults, addressing barriers to PE delivery in schools remains a public health priority.

## Introduction

In the face of the still growing pandemic of overweight and obesity, which results from a complex interaction of biological, behavioural, social, and environmental factors, exacerbated by sedentary behaviour and poor dietary habits [1,2], physical activity (PA), dietary and sedentary behaviours, sleep, and other individual and contextual factors contribute to energy balance and cardiometabolic health [1,3]. In this context, regular PA is a potent countermeasure to distinct physical inactivity (insufficient engagement in moderate- to vigorous-intensity physical activity) and sedentary behaviour (prolonged sitting or lying down, irrespective of PA levels). This is especially true if regular physical activity habits are established early in childhood, thereby promoting a healthy lifestyle in later adulthood [4]. In France, recent cohort studies indicated that only 51% of boys and 33% of girls aged 6–17 years met the World Health Organization (WHO) recommendations for physical activity [5], which was confirmed by recent cardiorespiratory fitness studies in children [6]. The main factors influencing the PA level of children aged from 3 to 11 years, are numerous and sometimes difficult to quantify, due to the lack of large-scale studies. Some of them have nevertheless been highlighted in specific studies, such as the parental PA level [7], their support for children’s PA habits [8], the mode of transport from home to school [9], the oppoertunity to spend leisure time outside of home [10] and the participation in a sports club [11].

However, the pandemic of physical inactivity and sedentary lifestyle makes these requirements difficult to meet [12]. This raises the question of the contribution of primary school in the PA level at this sensitive age (3–11 years), as primary school is the place where children spend most of their physically active time [13]. Interest in physical education (PE) may also be related to pupils’ attachment to school, teachers, and peer [14]. Hence, apart from recess breaks, PE is the only compulsory time in the curriculum schedule when children engage in locomotor activity. French national programmes require a weekly PE duration of 3 hour in elementary school and a minimum of 2 hours and 30 minutes in preschool [15]. However, in practise, the teacher remains solelyresponsible for the pedagogical progression. He or she may therefore be influenced by various constraints: for instance, a parliamentary report pointed out the “pressure” exerted by French language and mathematics curricula, both considered as a high priority by the authorities, thereby relegating other subjects, such as PE, to a secondary position [16]. Nevertheless, this single reason, which is not based on field surveys, might be insufficient to explain why the mean weekly PE duration was under 2 hours 15 minutes instead of the mandatory 3 hours. More recently, a study based on online questionnaires sent to primary teachers (PT) reported that only 11.7% of elementary classes met the required PE duration, vs. 82% in preschool, highlighting a marked discrepancy between these two sectors [17]. In this study, conducted on a limited sample, the PE teaching duration was positively correlated, among other factors, with years of experience, being a male PT and a sense of self-efficacy. Given the scarcity of research on barriers encountered by teachers and on other teacher- and school-related factors, the present study aims to assess on a larger scale, and in several departments across France, the mean weekly PE duration and to identify potential factors influencing it.

## Materials and methods

### Participants

This study was carried out from 12/3/2021–15/6/2024 in six French departments, following the gradual and progressive implementation of the survey across participating educational authorities: Pas-de-Calais (62), Nord (59), Vienne (86), Val-de-Marne (94), Seine-Saint-Denis (93) and Seine-et-Marne (77). Primary teachers, all adults, from 6 198 primary schools (2035 preschools and 4163 elementary schools) were contacted by email, sent from pedagogical advisor for primary schools, inspector of National Education or departmental services of the Ministry of National Education, and invited to take part in a study and complete an online questionnaire. Information on the purpose of the study and the content of the questionnaire were communicated to school principals, who were asked to pass this information onto their colleagues, along with a weblink to the questionnaire.

The study was approved by each departmental authorities of the French Ministry of Education before being distributed to primary teachers. The questionnaire was performed in accordance with relevant guidelines/regulations and with the Declaration of Helsinki [18]. Informed consent was documented as follows: the questionnaire's landing page explicitly informed participants, before any data were collected, that (i) the study was conducted in the context of academic scientific research, (ii) no personally identifiable data would be collected (no name, surname, or other identifying information was requested, and no information on racial, ethnic, political, religious, sexual, health, biometric or genetic background, nor social security number, was asked), (iii) participation was entirely voluntary, and (iv) data would be managed and analysed in accordance with French data-protection regulations (RGPD, Règlement Général sur la Protection des Données). Because the questionnaire was anonymous by design, no written or electronically signed consent form linked to participants’ identity could be collected without compromising that anonymity; consistent with standard practice for anonymous online surveys, and as approved by the departmental education authorities, consent was therefore obtained implicitly: teachers who, after reading this information page, voluntarily chose to proceed to and submit the questionnaire were considered to have given their informed consent to participate and to the use of their anonymous responses for research purposes. No response could be recorded without this voluntary act of proceeding past the information page and submitting the form.

### Sampling and response rate

The six participating departments were selected through an opportunity-based approach: they were progressively included over the study period (March 2021 to June 2024) as local partnerships were established with pedagogical advisors, inspectors, or departmental services of the Ministry of National Education who agreed to support and relay the survey in their department. The six-department sample was therefore primarily determined by the availability and willingness of local academic authorities to collaborate, rather than by a specified sampling frame designed to be representative of France as a whole. Within each department, the survey followed a three-step distribution: (i) an initial email including information on the study and a weblink to the questionnaire, was sent by the academic investigator to the pedagogical advisors, inspector, or departmental services, for approval and authorisation of diffusion; (ii) these information and weblink were transmitted by the aforementioned authorities to primary school principals; (iii) principals were then asked to relay this invitation to the teachers in their school. Because the questionnaire was not sent directly to individually identified teachers but distributed through this intermediary chain, the exact number of teachers who actually received the invitation could not be tracked or verified.

As a result, the participation rate reported in the “Results” section must be understood as a population-based rate. For each department, the numerator was the number of valid, retained questionnaire responses (after exclusion of input errors, duplicates and aberrant answers, see “Results” section), and the denominator was the total number of primary school teachers officially registered in that department, drawn from Ministry of National Education staffing statistics [19]. Because the denominator includes an unknown proportion of teachers or school principals who may not have received the invitation, this population-based rate is best interpreted as a conservative estimate of the real response rate among teachers who were actually exposed to the invitation; the latter cannot be calculated from the available data.

### Survey design

Designed by academics (sociology, educational sciences, health/physiology) and educational professionals (primary teacher, pedagogical adviser, administrators), the survey was conceived to cover various fields: personal et professional background, PA habits, geographic and socioeconomic context of school, specific school and class characteristics, weekly duration of PE teaching, professional skills and appetence in PE teaching. All items were multiple choice questions for fewer error inputs and more convenient statistical analyses.

#### Personal and professional background.

This section includes information about sex, age, seniority in teaching, professional status (tenured, full-time, intern, replacement squad, …) and initial university cursus. For further statistical analyses, teachers’ age and seniority were categorized in 5-year wide intervals (0–5 years, 5–10 years, 10–15 years, …).

#### Habits in physical activity.

In this section, participants were asked about their PA frequency (from ‘Never’ to ‘3~4 times per week), intensity (from ‘Sedentary, i.e., less than 30 min of slow walk per day’ to ‘Very active, i.e., 3 hrs fast walk or 1 hr fast walk + 1r vigorous activity’) and if they were a member of a sport club / association.

#### Geographic and socioeconomic context.

This section collected information about school urban environment (‘urban’, ‘suburban’ or ‘rural’), administrative socioeconomic status (‘none’, ‘low-’ or ‘high-priority educational network’ known as REP and REP + , respectively), number of children in school and in class, the presence of a school sport association (USEP, ‘Union sportive de l'enseignement du premier degré’) and the implementation of a national PA initiative (“APQ, Activité Physique Quotidienne”: Daily Physical Activity).

#### PE: Weekly duration.

This section includes only two items: one about the mean whole duration for PE in the class planning and one about the “peri-PE”, included in the first item, but dedicated to tasks that are not explicitly PE, such as transportation or walk to gymnasium, changing room, etc.

#### Professional skills and appetence in PE teaching.

The participants were asked to provide detailed information about grade(s) in their class (from ‘TPS: Toute Petite Section, 2~3 yrs old’ to ‘CM2: Cours Moyen 2, 10~11 yrs old’ and ‘inclusive classes’), the presence of pupils with specific needs, and the obstacles which keep the PT from meeting the programme requirements (distant location, quantity/quality of sport equipment, quantity/quality of infrastructures, weather condition, feeling of incompetence, lack of time for French/maths teaching, group management during PE lessons, safety reasons, lack of appetence for PE teaching), the intervention modalities (alone or with another person: school colleague, PE teacher from middle/high school, club coach, town employee, volunteer, etc.), the appetence for PE teaching, the feeling of competence in PE teaching (from ‘I feel very competent’ to ‘I do not feel competent’) and the specific training in PE teaching in primary school.

### Statistical analyses

Quantitative variables (various durations, PT age and seniority, number of pupils) were expressed in mean ± standard deviation (mean ± SD), and their normalities were tested by a Shapiro-Wilk test. Qualitative variables were quantified by categories such as department, sex, professional status, etc.

To unveil the variables influencing the PE duration, we first performed a stepwise linear regression to select relevant numerical and categorical variables explaining variations in PE duration. Then, we implement these variables into a multivariable regression model, to identify their statistical significance.

Tukey post hoc tests, effect size (standardized β and Cohen’s f^2^ for stepwise regression, η^2^ for multivariable regression and Cohen’s d for post hoc tests), confidence intervals (95%) and adjusted coefficients (r^2^, p) were computed when applicable.

Statistical analyses were performed with Jamovi 2.6.19.0 (Open-source software, Sydney, Australia).

The statistical signiﬁcance was set at p < 0.05.

## Results

Seventy-three input errors were excluded from the initial collected data: duplicate entries (n = 21) and aberrant answers regarding age (> 70 yrs old, n = 7), seniority (> 50 yrs, n = 9), number of pupils in school (>600, n = 6), number of pupils in class (>45, n = 9), PE duration (>6h, n = 12), peri-PE duration (>4h, n = 9). Then, 1967 answers from the 6 departments were retained for further analysis (Table 1). Teachers ‘age and seniority are shown in Table 2; their initial university training in Fig 1; their sport habits (frequency, club license) in Table 2; the socio-demographic context (urban context, education network) in Table 2; the specific school data (number of classes, number of pupils per school/class/level, single- or multigrade, presence of a sport school association, effective implementation of APQ, presence of external contractor as PE teacher) in Table 3; the weekly PE durations in Table 4; primary obstacles in Fig 2.

**Table 1:** Numbers of participating primary teachers and their percentages from the total number, by departments.

Department	Nord (59)	Pas-de-Calais (62)	Seine-et-Marne (77)	Vienne (86)	Seine-Saint-Denis (93)	Val-de-Marne (94)	Total
n (% of total) (M/F, %)	457 3.5% (23/77%)	241 3.1% (16/84%)	562 4.9% (14/86%)	205 10.5% (18/82%)	253 2.7% (15/85%)	249 3.5% (12/88%)	1967 3.9% (17/83%)

**Table 2:** Mean age and seniority of participating teachers, urban context and education network, frequency of physical activity and sport license.

	20~30	30~40	40~50	50~60	> 60
Age (%)	7%	21%	44%	24%	3%
	0~10	10~20	20~30	30~40	> 40
Professional seniority (%)	25%	32%	30%	10%	1%
	Rural		Suburban	Urban	
Urban context (%)	29%		14%	57%	
High-priority education network (REP/REP+)				25%	
	Never	Rarely	3 to 4 times per month	1~2 times per week	3~4 times per week
Personal physical activity: frequency (%)	4%	18%	7%	47%	23%
Sport license (club, association)					38%

**Table 3:** School context: number of classes, number of pupils per school and per class, number of pupils per cycle and per level (Cycle 1: TPS Toute Petite Section, MS Moyenne Section and GS Grande Section; Cycle 2: CP Cours Préparatoire, CE1 Cours Elémentaire 1 and CE2 Cours Elémentaire 2; Cycle 3: CM1 Cours Moyen 1 and CM2 Cours Moyen 2), single and multigrade class, sport school association, APQ implementation, external contractor and affinity for PE teaching.

Number of classes per school (percentage)	<5 classes	5~10 classes	10~15 classes	>15 classes						
	43%	24%	23%	10%						
Total number of pupils per school (percentage)	<50	50~100	100~200	200~300	300~400	>400				
	7%	17%	40%	22%	10%	4%				
Number of pupils per class (percentage)	<15	15~20	20~25	25~30	>30					
	7%	11%	49%	31%	2%					
Cycle	Cycle 1			Cycle 2			Cycle 3			Specific needs (6~11)
Class (pupils age)	TPS (2~3)	PS (3~4)	MS (4~5)	GS (5~6)	CP (6~7)	CE1 (7~8)	CE2 (8~9)	CM1 (9~10)	CM2 (10~11)	
Number of pupils per level (percentage)	2%	16%	9%	10%	10%	10%	12%	13%	18%	< 1%
	Single-grade class			Multigrade class						
	Cycle 1	Cycle 2	Cycle 3	Cycle 1	Cycle 2	Cycle 3	Cycle 1/2	Cycle 2/3		
	18%	23%	17%	17%	8%	9%	2%	7%		
Existence of a sport school association (‘USEP’)		13%								
APQ implementation		55%								
External contractor in PE	No	60%								
	Yes	40%								
Affinity for PE teaching	No	22%								
	Yes	78%								

**Table 4:** Mean (± standard deviation and confidence interval) durations of physical education, per department, for pre- and elementary school and per cycle.

Preschool (Cycle 1)	Nord (59)	2h21min ± 1h3min [2h11min; 2h31min]
Pas-de-Calais (62)	2h23min ± 55 min [2h10min; 2h35min]
Seine-et-Marne (77)	2h20min ± 1h1min [2h12min; 2h29min]
Vienne (86)	3h04min ± 1h5min [2h47min; 3h20min]
Seine-Saint-Denis (93)	2h10min ± 1h7min [1h55min; 2h25min]
Val-de-Marne (94)	2h20min ± 1h13min [2h5min; 2h35min]
Elementary school (Cycles 2 & 3)	Nord (59)	2h46min ± 48 min [2h40min; 2h51min]
	Pas-de-Calais (62)	2h13min ± 41 min [2h7min; 2h19min]
	Seine-et-Marne (77)	2h32min ± 54 min [2h27min; 2h38min]
	Vienne (86)	2h34min ± 46 min [2h26min; 2h41min]
	Seine-Saint-Denis (93)	2h10min ± 49 min [2h2min; 2h17min]
	Val-de-Marne (94)	2h17min ± 56 min [2h8min; 2h26min]

**Fig 1:**
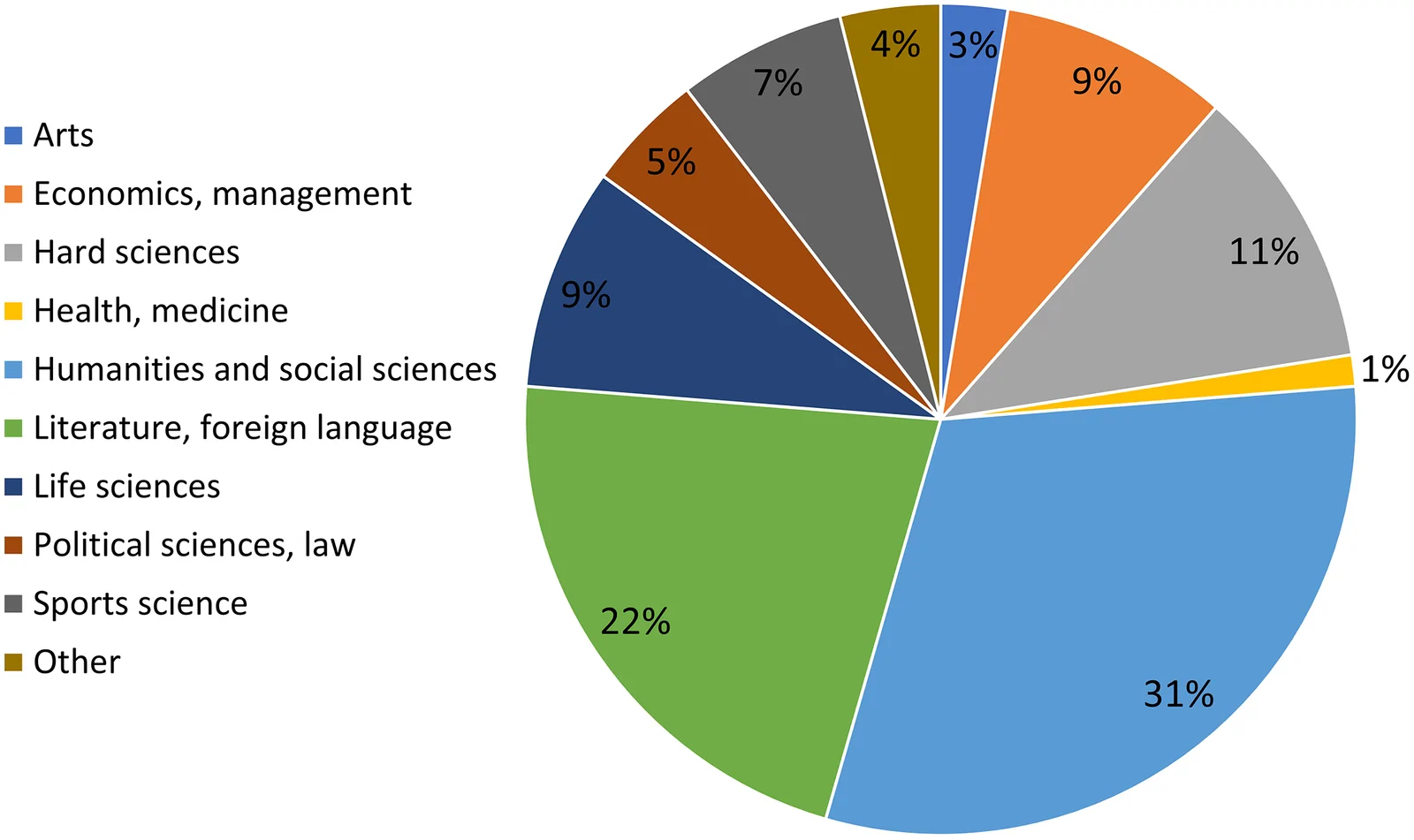
University education of participating teachers.

**Fig 2:**
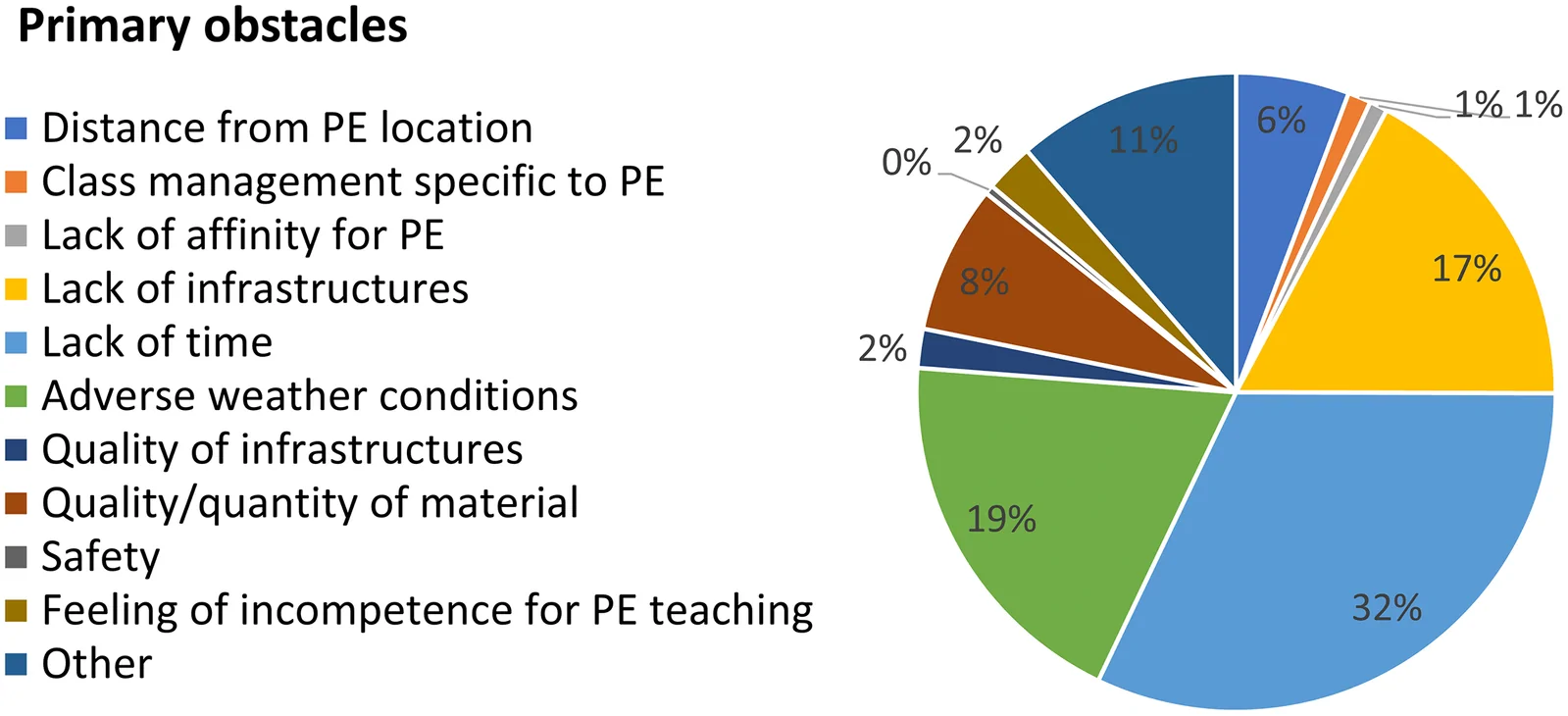
Primary obstacles reported by participating teachers.

We compared variables of the present sample with available ones in larger cohort (regional or national, according availability), from official public data [19]. First, our sample was composed of 83,4% women and 16,6% men, which is close to national numbers (86,2% and 13,8%, respectively). The same goes for age (43.9 ± 8.7 for women and 44.5 ± 8.9 yrs old for men vs. 43,3 and 44,8 yrs old, respectively), ratio between number of teachers in pre- and elementary schools (34,4% and 65,6% vs. 34,5% and 65,5%, respectively). Other variables were lacking or being expressed differently, making subsequent comparisons impossible.

Nine predictor variables, were selected after the stepwise regression: four numerical (seniority, standardized β = 0.092; number of pupils in the class, β = −0.053; peri-PE duration, β = 0.421; number of declared obstacles, β = −0.107) and five categorical (urban context, inclusion of the school in a REP/REP+ area, implementation of APQ, multigrade class and the feeling of competence in teaching PE). These variables were then entered into the multivariable linear regression model, for which unstandardized coefficients, 95% confidence intervals, partial η² and overall model fit are reported in Table 5. In addition to these nine retained predictors, other variables collected in the survey (including teachers’ sex, age, initial university cursus, initial and continuing PE-specific training, personal physical-activity habits, department, presence of a sport school association, and presence of an external contractor in PE) were also evaluated by the stepwise procedure but were not retained in the final model, indicating no significant independent association with PE duration. Because several candidate variables are conceptually or empirically related to one another (e.g., age and seniority; or urban context, school size, priority-education status, external-contractor presence, and department), we deliberately used this multivariable approach so that each association reported in Table 5 is adjusted for the other selected predictors, providing an estimate of the independent association between each variable and PE duration rather than a marginal (unadjusted) one.

**Table 5:** Multivariable linear regression model of weekly PE duration (hours), showing unstandardized coefficients, standard errors, 95% confidence intervals, partial η^2^ and overall model fit (N = 1,960).

Predictors	b	SE	95% CI	Partial η^2^	p
Intercept	2.432	0.035	[2.363, 2.501]	–	<0.001
Seniority	0.009	0.002	[0.005, 0.013]	0.010	<0.001
Number of pupils per class	–0.011	0.005	[–0.020, –0.001]	0.003	0.024
Peri-PE duration	1.068	0.050	[0.970, 1.165]	0.191	<0.001
Declared obstacles (count)	–0.080	0.016	[–0.110, –0.049]	0.013	<0.001
Urban context (ref: Rural)				0.004	
Suburban	0.060	0.062	[–0.061, 0.181]		0.591
Urban	–0.093	0.048	[–0.205, 0.020]		0.129
School sector (none or REP/REP+)	−0.080	0.050	[–0.177, 0.018]	0.001	0.109
APQ implementation (APQ or no APQ)	0.299	0.037	[0.225, 0.372]	0.032	<0.001
Class type (single or multigrade)	0.101	0.040	[0.023, 0.179]	0.003	0.011
Feeling of competence in PE (ref: Not competent)				0.020	
Slightly competent	–0.005	0.118	[–0.237, –0.226]		0.964
Neither competent nor incompetent	0.172	0.112	[–0.048, 0.392]		0.125
Fairly competent	0.306	0.114	[0.082, 0.530]		0.007
Very competent	0.450	0.136	[0.184, 0.717]		<0.001

*Overall model fit: R^2^ = 0.268; adjusted R^2^ = 0.263; F(13, 1960) = 55.1, p < 0.001; N = 1,960.*

Although bivariate analysis showed several association between PE duration and several variables, such as sex and age for example, these relationships did not persist in the multivariable model, suggesting that they were not independent of other covariates.

From all the aforementioned selected variables, the multivariable linear regression model highlighted the following ones as significantly influencing PE duration (see Table 5): seniority, number of pupils in class, peri-PE duration, urban context (p = 0.035, η*^2^* = 0.004), implementation of APQ, multigrade class and the feeling of competence (p < 0.001, η*^2^* = 0.031).

More precisely, a longer seniority was associated with a longer PE duration (p < 0.001) but a higher number of pupils in the class was associated with a shorter PE duration (p = 0.024). A longer PE duration is associated with a longer peri-EPS (p < 0.001). As for urban context, there was no difference between zones, except for PE duration being longer in suburban area than in urban ones (p = 0.027, d = 0.057). Implementing APQ and teaching in a multigrade class increase PE duration (p < 0.001, d = 0.038 and p = 0.038, d = 0.042, respectively). Finally, teachers stating higher competence in PE teaching reported longer PE durations; for instance, feeling ‘very’ or ‘enough competent’ has a stronger effect on PE duration than ‘not competent’ (p < 0.001, d = 0.135 and p = 0.008, d = 0.114, respectively), than ‘not very competent’ (p < 0.001, d = 0.095 and p < 0.001, d = 0.060, respectively) and than ‘neither competent nor incompetent’ (p = 0.003, d = 0.085 and p = 0.003, d = 0.042, respectively).

## Discussion

This study was conducted in several French departments (Table 1) and relied on teachers’ voluntary will responses, although French primary school teachers face substantial daily psychological and workload daily pressures, arising from teaching (including classroom management), lesson preparation, marking, teamwork and meetings, and administrative duties [20], while salaries and the attractiveness of the profession are lower than in other European countries [21]. Given these constraints, teachers’ participation in this voluntary survey can nevertheless be viewed as a positive aspect of the study, despite the apparently low overall response rate (below 4%, Table 1).

Consistent with previous studies and government reports [16,17,22,23], our findings indicate that both preschool and elementary school teachers reported PE durations below the curriculum requirements (Table 4). However, the shortfall relative the official requirements was smaller in preschool (< 5%) than in elementary school (−18%). This may be due to the availibility, in most preschools, of a dedicated PE space or room with a motor activity circuit that can be used daily at scheduled times, over extended periods (6–12 weeks, depending on the teaching programme), in accordance with official curriculum recommendations [15]. In contrast, PE equipent in elementary schools usually has to be set up for each lesson. This pedagogic time efficiency by preschool timetables and permanent equipment arrangements has previously been observed in France [17] and in USA [24]. This may also be related to peri-PE time (transportation to gymnasium, changing clothes, …), which is shorter in preschool than in elementary school. When preschool PE lessons in preschool take place every day at the same dedicated place within the school, teachers can save a substantial amount of travel and setup time and allocate it to the PE lesson itself, including actual physical activity. Our data also show that longer PE duration is associated with more time spent on peri-PE tasks (transportation, changing room, …). Previous reports [16] identified these tasks a factor associated with shorter overall PE duration. Paradoxxically, our findings show the opposite association, for which we found no comparable evidence in the current literature: we hypothesize that teachers may compensate for longer peri-PE duration by extending the time devoted to physical activity.

Beyond these findings, our adjusted statistic model also indicated that neither teachers’ sex nor their personal sports habits were associated with time spent in teaching PE, in contrast to the limited previous research [25]. Previous studies has shown that men participate in sports more than women [26], and that PE teaching may be perceived as a “masculine” activity [27]; in our sample, however, men and women did not differ in their personnal PA habits or in the duration of PE teaching.

Longer PE duration was associated with greater seniority (Table 2), but not with age. One plausible explanation, not directly assessed by our questionnaire, is that the accumulated teaching experience improves efficiency in organising and delivering PE [17,28]. Unexpectedly, neither initial professional training in PE nor continuing professional development was associated with PE duration. Evidence on this topicis limited: one French study reported a positive association with the perceived quality, rather than duration, of initial and continuing PE training [17], whereas an English study reported contrasting finding for both [29]. Our findings are consistent with the latter, although none of these studies examined training content, which may play a key-role in teachers’ engagementwith PE.

A related finding is that teachers’ initial university eduaction (Fig 1) was not associated with PE duration, in contrast to another recent study [17]. A background in sports sciences might be expected to facilitate and encourage PE teaching, particularly because sports science curricula includes specific content on PE teaching. However, our data does not support this hypothesis: teachers with a background in sport and physical activity, like those with other educational backgrounds, did not meet the required the PE teaching. Despite their specific skills, we can conjecture that these teachers meet the same general barriers as to meeting the time requirements.

At an individual level, the feeling of competence for PE teaching was associated with PE duration. This perceived competence, which is linked to seniority, was associated with more time spent teaching PE: teachers who felt more confident in their teaching skills reported longer PE durations, consistent with previous studies [17,30]. Recent evidence also suggests that the quality of working life may be related to PE teachers’ motivation to teach [31]. Although initial professional training in PE, distinct from initial university education, can help build the feeling of competence [32], we did not observe an association between such training and PE duration. Instead, PE duration was strongly associated with practical teaching experience (“field experience”) rather than PE-specific training, in contrast to some previous studies examining the perceived quality of training [17].

Beyond teachers’ personal backgrounds, geographic, socioeconomic and school contexts may also be relevant. First, PE duration varied with urban context (Table 2), with longer durations in suburban areas than in urban and rural areas. This pattern is broadly consistent with a ministerial report [16]. Although we did not test this hypothesis in our study, rural schools may offer more opportunities for outdoor PE, such as open-air activities [33], but lack certain sports facilities, particularly swimming pools, and therefore need to allocate funds into transportation to stadiums or pools. Such facilities may be more readily available and better funded in urban and suburban areas. Conversely, in densely populated areas, the existing facilities, especially swimming pools, may be oversubscribed, limiting some pupils’ access to appropriate PE instructions.

Moreover, although 40% of respondents reported working with an external contractor (Table 3), this variable was not an independent predictor of PE duration in our multivariable model. This raises a longstanding issue concerning these interventions. Primary school teachers are pedagogically responsible for delivering the entirety of the national curriculum, including PE, but they may delegate PE teaching to the external contractors, usually coaches from sports clubs or associations, or municipal staff. This can result in subject-specifing instruction resembling that provided in secondary schools, rather than the pedagogical approach recommended by the Ministry of Education [34].

Moreover, in high-priority education areas (REP/REP+), a large majority of primary school teachers mentioned additional difficulties in managing pupil that are linked to the low socioeconomic status of these areas [35]. These difficulties may be amplified outside the classroom, including PE lessons. Few studies examined the differences in PE duration between these schools and those outside high-priority areas; previous research suggests the aforementioned teaching difficulties in REP/REP+ schools are associated with shorter PE duration [25]. Nevertheless, we observed no such difference, suggesting that management issues related to both socioeconomic context and PE were not associated with PE duration in our sample. The reasons for this absence remain to be explored. Interviews with teachers working in REP/REP+ areas could help investigate the potential benefits of PE for pupils’ attention and concentration in other subjects, such as French language and mathematics [36,37].

Regarding school-level factors, larger class size was associated with shorter PE duration: although larger classes do not appear to affect overall academic outcomes, they do influence teachers’ classroom management practises [38], particularly by reducing opportunities for group work that:can benefit pupils with learning difficulties [39]. The intrinsic demand of PE, including outdoor settings, continuous movement, equipment use, noisy environment and safety requirements, may increase teachers’ reluctance to teach the subject [40]. Unexpectedly, the number of classes in a school was not associated with PE duration, despite the potential scheduling difficulties involved in sharing limited space. In the same manner, multigrade classes spent more time on PE than single-grade classes (Table 3). This is surprising, given the additional challenges of covering various curricula, differentiating and adaptating instruction, and managing pupil heterogeneity [41]. To our knowledge, no studies have specifically examined the benefits of PE in multigrade classes. However, the recognised psychosocial benefits effects of multigrade teaching, such as autonomy, cooperation and socialisation, may also apply to PE [42]. Also surprisingly, the presence of a school sports association (USEP, Table 3) was likewise not associated with longer PE duration, although USEP trainers and coaches are usually primary school teachers. As with personal sport habits, we had hypothesized that sports-related skills and affinity might transfer from USEP activities to PE teaching, but our data data do not support this hypothesis. Conversely, teachers who were not involved in USEP activities still reported similar PE durations to their counterparts, suggesting that affinity and involvement in related-sports activities were not associated with more time spent teaching PE.

Piloted from 2020 and subsequently mandated by legislation in 2022 [43], APQ (‘Activités Physiques Quotidiennes’, Daily Physical Activity) was implemented in French primary school as a public health measure to counteract deleterious effects of sedentary lifestyles. However, official texts distinguished APQ from PE and defines it as a complement to existing PE provision. Although APQ is compulsory under the national programmes, our study shows that only 55% of primary teachers of our sample reported implementing APQ in their timetables (Table 3). Our findings are in line with previous publications suggesting that primary school teachers may be reluctant to implement this imposed and insufficiently defined measure, and may combine it to Physical Education [44]. We designed our questionnaire to distinguish clearly PE and APQ, in accordance with the official French programmes. However, a Senate report pointed out the confusion over the terminology and the distinction between the two, leading a substantial number of teachers to overestimate PE duration by including APQ time [45]. The actual mean PE duration may therefore be lower than our estimate, although we could not verify this possibility.

Regarding the barriers to PE teaching reported by teachers (Fig 2), most authors pointed out, among others reasons, the lack of professional training, low self-confidence, difficulties in managing groups and insufficient infrastructures and equipment [17,23,29,40]. However, our findings did not fully confirm these associations, particularly for initial and continuing training. Given the diversity of reported barriers, we examinded their associations with PE duration using the number of stated barriers. A greater number of reported barriers were tightly associated with shorter PE duration. Lack of time was the most frequently reported barrier for both pre- and elementary school (Fig 2). This issue has been raised, although not quantified, in previous large-scale studies and reports: under institutional pressure [23,46,47], teachers prioritise French language and mathematics teaching over other subjects, including PE [16,48]. Weather conditions were the second most frequently reported barrier (Fig 2), an issue also poorly documented in the literature. We did not collect or analyse department-level meteorological data, and therefore could not determine whether day-to-day weather conditions contributed to differences in PE duration between departments (Tables 1 and 4). Further studies covering a wider range of French departments would help clarify the relationship weather conditions and PE duration.

## Limitations

Our study carries some limitations, intrinsic to this type of survey. First, a desirability bias of self-report could influence the declared PE duration, especially within an institutional context where teachers’ activities are regularly monitored; however, we clearly specified in the questionnaire presentation that it was strictly anonymous (no identification information were asked), that answers would be collected by academic researchers, and not administration representatives from the Ministry of National Education, so the participants could answer as freely and truly as possible. As an example, 45% of participants admitted not implementing APQ although it’s a compulsory subject in all levels. As for the reported PE duration, it was not possible to verify these data unless conducting a follow-up monitoring, on site, in every surveyed schools; as mentioned above, the questionnaire design aimed for participants to answer freely and truthfully. Second, the sampling and distribution procedure can create potential selection bias. The voluntary nature of participation could induce a self-selection bias, whereby teachers more interested in, or conversely more frustrated by, PE teaching may be over-represented among respondents: although 22% of participants declared having no affinity with PE teaching, our statistical multivariable model clearly ruled this parameter out, implying that there was no difference in PE duration between teachers keen on PE teaching and their counterparts. A distinct source of selection bias arises from the cascading distribution of the questionnaire: because the survey was relayed through educational inspectors and school principals rather than sent directly to individually identified teachers, we could not verify whether all primary teachers actually received the invitation, and principals more favourable to, or engaged with, PE may have been more likely to forward it to their colleague teacher.

Considering the high number of inspectors and school principals in each department (from 8 in Vienne to 40 in Seine-Saint-Denis, and from ~300 in Vienne to ~1550 in North, respectively), it was not possible to thoroughly check if all primary teachers actually received the questionnaire. This gatekeeper-dependent relay chain likely also contributes to the wide variation in department-level participation rates reported in Table 1 (2.7% to 10.5%): shorter or more direct relay chains, as in Vienne, may increase effective reach and therefore apparent participation, independently of teachers’ interest in PE, so that these department-level rates should not be read as indicators of differential engagement in PE across departments. This is a limitation that could be addressed in future studies, for example, by closely supervising (on site) the questionnaire administration or by using a sampling frame with individually identifiable participants; but this would require a much longer time to complete the study at a departmental scale and a tight organization to ensure participants’ anonymity. Reassuringly, comparison of our sample with official national demographic data showed close alignment on sex, age and the pre-/elementary school distribution, suggesting that gross demographic selection bias was limited. However, this comparison could not assess whether respondents differed from non-respondents on less visible characteristics, such as motivation or attitudes toward PE teaching, which are more directly relevant to the outcome under study and cannot be ruled out as a source of bias. Finally, this study was conducted in six French departments, selected according to the availability and willingness of local academic authorities to support the project rather than through random or stratified national sampling. Although these departments varied in geographic and socio-economic profile, the sample is not nationally representative; findings should therefore not be generalized to France as a whole, nor to other national contexts, without further confirmation in a broader or sampled cohort.

## Conclusion

In this sample of primary school teachers from six French departments, self-reported physical education teaching time did not meet the durations required by the national programmes, and was associated with teachers’ seniority, the urban context, number of pupils in class, the multigrade modality and the feeling of competence. Given the cross-sectional, self-report design and the limited geographical scope of the sample, these associations should not be interpreted as causal, and generalization beyond the surveyed departments should be made with caution. We are aware, given that this survey was completed solely on voluntary will in a very tight time schedule, that maybe most answering teachers showed an interest in PE teaching, and a wider survey would be relevant to confirm, tune or infirm our findings.

Physical education represents a potent and flexible opportunity for structured physical activity during childhood, and insufficient PE duration is one among several factors contributing to sedentary lifestyle and its long-term health consequences (obesity, diabetes mellitus, cardiovascular pathologies, etc.) [49]. Our study did not assess children’s health status, body composition or cardiovascular risk and whether improved PE provision can contribute to identifying (and supporting) children at health risk is an important question for future research.

This article only aims to describe, at a given time, the relationship between primary teachers and physical education, and what could be the factors weighing on it. We do not suggest any solution at this point, as potential propositions should be tested and analysed in further studies, knowing that numerous courses of actions on the field are plausible, going from engagement, motivation, initial and continuous training and pedagogic differentiation.
