# Supplementary material for: Duration and barriers of physical education teaching in primary schools in 6 French departments
Source: PLoS One. 2026 Nov 3;21(11):e0362195. doi: 10.1371/journal.pone.0362195 (PMC13730362; doi:10.1371/journal.pone.0362195)
Supplement: Supplementary file 1 [file pone.0362195.s001.pdf]

PLOS One

Physical Education in Primary schools: an overview of teaching practices in France

--Manuscript Draft--

**Manuscript Number:**	PONE-D-26-17765
**Article Type:**	Research Article
**Full Title:**	Physical Education in Primary schools: an overview of teaching practices in France
**Short Title:**	Physical Education in French Primary schools
**Corresponding Author:**	Eric Hermand, PhD<br>Universite Sorbonne Paris Nord - Campus de Bobigny<br>Bobigny, FRANCE
**Keywords:**	Physical Education; primary school; curriculum; Health
**Abstract:**	Physical activity is the primary defence against sedentary lifestyles leading to chronic diseases (obesity, type 2 diabetes). This study aimed to assess durations of Physical Education (PE) in several French departments and to identify the main obstacles to PE teaching. From 2021 to 2024, an online questionnaire was sent to primary teachers in six departments about their professional habits in teaching this subject. A total of 1,967 responses were collected and analysed: neither preschool (2h23min±1h04min) nor elementary school (2h28min±51min) teachers met the mandatory durations imposed by the Ministry of National Education. The main barriers identified were lack of time, inadequate infrastructure, and adverse weather conditions. Given the essential role of physical activity in the health of children and future adults, addressing these barriers in schools remains a public health priority.
**Order of Authors:**	Eric Hermand, PhD
	Serge Durali
	Hugues Rolan
	Laurence Munoz, PhD
	Thierry Pezé
	Valentin Smee
**Additional Information:**	
**Question**	**Response**
**Financial Disclosure**<br><br>Enter a financial disclosure statement that describes the sources of funding for the work included in this submission. Review the submission guidelines for detailed requirements. View published research articles from *PLOS ONE* for specific examples.<br><br>This statement is required for submission and **will appear in the published article** if the submission is accepted. Please make sure it is accurate.	The author(s) received no specific funding for this work.

Powered by Editorial Manager® and ProduXion Manager® from Aries Systems Corporation

**Funded studies**<br>Enter a statement with the following details:<br>• Initials of the authors who received each award<br>• Grant numbers awarded to each author<br>• The full name of each funder<br>• URL of each funder website<br>• Did the sponsors or funders play any role in the study design, data collection and analysis, decision to publish, or preparation of the manuscript?<br><br>Did you receive funding for this work?	
**Competing Interests**<br><br>Use the instructions below to enter a competing interest statement for this submission. On behalf of all authors, disclose any competing interests that could be perceived to bias this work—acknowledging all financial support and any other relevant financial or non-financial competing interests.<br><br>This statement is required for submission and **will appear in the published article** if the submission is accepted. Please make sure it is accurate and that any funding sources listed in your Funding Information later in the submission form are also declared in your Financial Disclosure statement.<br><br>View published research articles from *PLOS ONE* for specific examples.	The authors have declared that no competing interests exist.

**NO authors have competing interests**<br><br>Enter: *The authors have declared that no competing interests exist.*<br><br>**Authors with competing interests**<br><br>Enter competing interest details beginning with this statement:<br><br>*I have read the journal's policy and the authors of this manuscript have the following competing interests: [insert competing interests here]*<br><br>* typeset	
**Ethics Statement**<br><br>Enter an ethics statement for this submission. This statement is required if the study involved:<br><br>• Human participants<br>• Human specimens or tissue<br>• Vertebrate animals or cephalopods<br>• Vertebrate embryos or tissues<br>• Field research<br><br>Write "N/A" if the submission does not require an ethics statement.<br><br>General guidance is provided below. Consult the submission guidelines for detailed instructions. **Make sure that all information entered here is included in the Methods section of the manuscript.**	Regarding our methodology and ethics, our questionnaire was completely anonymous (no name/surname were asked) and did not ask any information about racial/ethnic/politic/religious/sexual/health/ biometric/genetic background, neither social security number. Therefore, as per national ethic regulations, the only necessary authorization was from regional offices of Ministry of Education, which we all obtained (it was a mandatory passage to send the questionnaire out to teachers anyway).<br><br>The study was approved by each departmental authorities of the French Ministry of Education and was performed in accordance with relevant guidelines/regulations and with the Declaration of Helsinki. The participation was voluntary and the questionnaire clearly stated the objectives and modalities of this academic research. All participants provided informed consent.

Format for specific study types

Human Subject Research (involving human participants and/or tissue)

- Give the name of the institutional review board or ethics committee that approved the study
- Include the approval number and/or a statement indicating approval of this research
- Indicate the form of consent obtained (written/oral) or the reason that consent was not obtained (e.g. the data were analyzed anonymously)

Animal Research (involving vertebrate animals, embryos or tissues)

- Provide the name of the Institutional Animal Care and Use Committee (IACUC) or other relevant ethics board that reviewed the study protocol, and indicate whether they approved this research or granted a formal waiver of ethical approval
- Include an approval number if one was obtained
- If the study involved *non-human primates*, add *additional details* about animal welfare and steps taken to ameliorate suffering
- If anesthesia, euthanasia, or any kind of animal sacrifice is part of the study, include briefly which substances and/or methods were applied

Field Research

Include the following details if this study involves the collection of plant, animal, or other materials from a natural setting:

- Field permit number
- Name of the institution or relevant body that granted permission

Data Availability

Before publication, Authors are required to make fully available and without restriction all data underlying their findings. Sharing the data underlying your findings increases the visibility, credibility, and impact of your work, while supporting transparency and future scientific discovery. Please see our PLOS Data Policy page for detailed information on this policy.</p>

A Data Availability

This study was performed under the supervision and agreement of Academic departmental authorities.
The data that support the findings of this study are available from the corresponding author, Dr. Eric Hermand, upon reasonable request

Powered by Editorial Manager® and ProduXion Manager® from Aries Systems Corporation

Statement, detailing where the data can be accessed, is required at first submission. Insert your Data Availability Statement in the box below.</p><br><br>Please see the data reporting of our submission guidelines for instructions on what you need to include in your Data Availability Statement. Examples of acceptable Data Availability Statements are included here for reference:</p><br> <br> - The minimal data set [and accompanying code, where generated] is available at [repository name] via [DOI hyperlink] <br> - No data was generated by this study. The following existing data sources were used: [dataset name] from [dataset location] available via [web address or DOI hyperlink]. <br> - For Study Protocols: No datasets were generated or analysed during the current study. All relevant data from this study will be made available upon study completion. <br> <br><br><i>PLOS allows rare exemptions to address legal and ethical concerns. If you have legal or ethical restrictions, please use the box below to detail these in full sentences for the Journal team to consider.</i></p><br><br>This statement will be typeset if the manuscript is accepted for publication.</p>	

Powered by Editorial Manager® and ProduXion Manager® from Aries Systems Corporation

Manuscript

Click here to access/download;Manuscript;PrimarySchool_Manuscript_v2.d

Physical Education in Primary schools: an overview of teaching practices in France

Eric Hermand[1]*, Serge Durali[2], Hugues Rolan[2], Laurence Munoz[3], Thierry Pezé[3], Valentin Smee[4]

[1]*Université Sorbonne Paris Nord, Hypoxie et Poumon, H&P, INSERM, U 1272, F-93000, Bobigny, France*

[2]*Université Sorbonne Paris Nord, Département STAPS, Bobigny, France*

[3]*Université Littoral Côte d'Opale, Université Artois, Université Lille, CHU Lille, ULR 7369 - URePSSS - Unité de Recherche Pluridisciplinaire Sport Santé Société, F-59140 Dunkerque, France*

[4]*Université Littoral Côte d'Opale, Calais, France.*

*corresponding author

E-mail: eric.hermand@univ-paris13.fr (EH)

Abstract

Physical activity is the primary defence against sedentary lifestyles leading to chronic diseases (obesity, type 2 diabetes). This study aimed to assess durations of Physical Education (PE) in several French departments and to identify the main obstacles to PE teaching. From 2021 to 2024, an online questionnaire was sent to primary teachers in six departments about their professional habits in teaching this subject. A total of 1,967 responses were collected and analysed: neither preschool (2h23min±1h04min) nor elementary school (2h28min±51min) teachers met the mandatory durations imposed by the Ministry of National Education. The main barriers identified were lack of time, inadequate infrastructure, and adverse weather conditions. Given the essential role of physical activity in the health of children and future adults, addressing these barriers in schools remains a public health priority.

Keywords: physical education; primary school; curriculum; health

Introduction

In the face of the still growing pandemic of overweight and obesity, mainly caused by sedentary behaviour and poor dietary habits [1], physical activity (PA) remains the first and major countermeasure to reduce its deleterious effects on global health [2]. This is especially true if habits of regular physical activity are settled from early childhood at young age, hence promoting a healthy physical lifestyle during later adulthood [3]. In France, recent cohort studies pointed out that only 51% of boys and 33% of girls aged 6 to 17 years old were reaching the World Health Organisation (WHO) recommendations for physical activity [4], which was confirmed by recent cardiorespiratory fitness studies in children [5]. Regarding this matter, the main factors influencing the PA level of young children, from 3 to 11 years-old, are multiple and sometimes difficult to quantify due to the

lack of large-scale studies. Some of them were however highlighted in specific protocols, such as the parents' PA level [6], their support in child's PA habits [7], the mode of transport from home to school [8], the possibility to spend leisure time outside of home [9] and the participation in a sports club [10].

However, the pandemic of physical inactivity and sedentary lifestyle make these requirements problematic to reach [11]. This raises the question of the contribution of primary school in the PA level at this sensitive age, between 3 and 11 years old, as primary school is the place where children are spending most of their physically active time [12]. Hence, except recess breaks, Physical Education (PE) is the only mandatory time in curriculum schedule where children are involved into motor locomotion. French national programs impose a weekly 3-hour PE duration in elementary school and a minimum 2 hours and 30 minutes in preschool [13]. However, professionally speaking, the teacher remains the only person in charge of the pedagogic progression. Therefore, he/she may be influenced by various constraints: for instance, a parliamentary report pointed out the "pressure" exerted by French language and mathematic curriculums, both considered as high-priority by authorities, hence relegating other disciplines, such as PE, to a secondary level [14]. Nevertheless, this sole reason, not based on field surveys, might be insufficient to explain why the mean weekly duration of PE was under 2 hours and 15 minutes instead of the mandatory 3 hours. More recently, a study constructed upon online questionaries sent to primary teachers (PT) reported that only 11.7% of elementary classes met the required PE duration, vs. 82% in preschool, highlighting a dramatic discrepancy between these two sectors [15]. In this study, performed on a limited sample, the PE teaching duration was positively correlated, among other factors, to experience years, being a male PT and the sense of self-efficacy. In the light of scarce research in this areas, the present study aims to assess on a larger scale, in several departments across the French territory, the weekly mean PE duration and to unveil potential factors influencing it.

Materials and methods

Participants

This study was carried out from 12/3/2021 to 15/6/2024 in six French departments: Pas-de-Calais (62), Nord (59), Vienne (86), Val-de-Marne (94), Seine-Saint-Denis (93) and Seine-et-Marne (77). PE teachers, all adults, from 6 198 primary schools (2035 preschools and 4163 elementary schools) were contacted by email, sent from pedagogical advisor for primary school, inspector of National Education or departmental services of the Ministry of National Education, and invited to take part in a study and complete an online questionnaire. Information on the purpose of the study and the content of the questionnaire were communicated to school principals, who were asked to pass this information onto their colleagues, along with a weblink to the questionnaire.

The questionnaire was entirely anonymous (no name or surname were asked) and did not ask any information about racial, ethnic, politic, religious, sexual, health, biometric and genetic background, neither social security number. No given information could allow the identification of participants in any way. The study was approved by each departmental authorities of the French Ministry of Education and was performed in accordance with relevant guidelines/regulations and with the Declaration of Helsinki [16]. The participation was voluntary and the questionnaire clearly stated the objectives and modalities of this academic research. All participants were provided explicit informed consent in the questionnaire presentation.

Survey design

The survey was conceived to cover various fields: personal et professional background, PA habits, geographic and socioeconomic context of school, specific school and class characteristics, weekly duration of PE teaching, professional skills and appetence in PE teaching. All items were multiple choice questions for fewer error inputs and more convenient statistical analyses.

Personal and professional background

This section includes information about sex, age, seniority in teaching, professional status (tenured, full-time, intern, replacement squad, …) and initial university cursus. For further statistical analyses, teachers' age and seniority were categorized in 5-year wide intervals (0-5 years, 5-10 years, 10-15 years, …).

Habits in Physical Activity

In this section, participants were asked about their PA frequency (from 'Never' to '3~4 times per week), intensity (from 'Sedentary, i.e. less than 30 min of slow walk per day' to 'Very active, i.e. 3 hrs fast walk or 1 hr fast walk + 1r vigorous activity') and if they were a member of a sport club / association.

Geographic and socioeconomic context

This section collected information about school urban environment ('urban', 'suburban' or 'rural'), administrative socioeconomic status ('none', 'low-' or 'high-priority educational network'), number of children in school and in class, the presence of a school sport association (USEP, 'Union sportive de l'enseignement du premier degré') and the implementation of a national PA initiative ("APQ, Activité Physique Quotidienne": Daily Physical Activity).

PE: weekly duration

This section includes only two items: one about the mean whole duration for PE in the class planning and one about the "peri-PE", included in the first item, but dedicated to tasks that are not explicitly PE, such as transportation or walk to gymnasium, changing room, etc.

Professional skills and appetence in PE teaching

The participants were asked to provide detailed information about grade(s) in their class (from 'TPS: Toute Petite Section, 2~3 yrs old' to 'CM2: Cours Moyen 2, 10~11 yrs old' and 'inclusive classes'), the presence of pupils with specific needs, and the potential reasons which keep the PT from meeting the program requirements (distant location, quantity/quality of sport equipment, quantity/quality of infrastructures, weather condition, feeling of incompetence, lack of time for French/maths teaching, group management during PE lessons, safety reasons, lack of appetence for PE teaching), the intervention modalities (alone or with another person : school colleague, PE teacher from middle/high school, club coach, town employee, volunteer, etc.), the appetence for PE teaching, the feeling of competence in PE teaching (from 'I feel very competent' to 'I do not feel competent') and the specific training in PE teaching in primary school.

Statistical analyses

Quantitative variables (various durations, PT age and seniority, number of pupils) were expressed in mean ± standard deviation (mean ± SD). Other qualitative variables were quantified by categories such as department, sex, professional status, etc.

Data normality was tested by a Shapiro-Wilk test and potential associations between variables were assessed using either Chi-square, Spearman or Kruskall-Wallis tests. Statistical analyses were performed with Jamovi 2.6.19.0 (Open-source software, Sydney, Australia).

The statistical significance was set at $p < 0.05$.

Results

After a clean of outliers (error of input entries, duplicates), 1967 answers from the 6 departments were retained for further analysis (table 1). Teachers'age and seniority are shown in table 2; their initial university training in figure 1; their sport habits (frequency, club license) in table 2; the socio-

demographic context (urban context, education network) in table 2; the specific school data (number of classes, number of pupils per school/class/level, single- or multigrade, presence of a sport school association, effective implementation of APQ, presence of external contractor as PE teacher) in table 3; the weekly PE durations in table 4; primary obstacles in figure 2.

Table 1. Numbers of participating primary teachers and their percentages from the total number, by departments.

Department	Nord (59)	Pas-de-Calais (62)	Seine-et-Marne (77)	Vienne (86)	Seine-Saint-Denis (93)	Val-de-Marne (94)	Total
n<br>(% of total)<br>(M/F, %)	457<br>3.5%<br>(23/77%)	241<br>3.1%<br>(16/84%)	562<br>4.9%<br>(14/86%)	205<br>10.5%<br>(18/82%)	253<br>2.7%<br>(15/85%)	249<br>3.5%<br>(12/88%)	1967<br>3.9%<br>(17/83%)

Table 2. Mean age and seniority of participating teachers, urban context and education network, frequency of physical activity and sport license.

<table>
<tr><td></td><td>20~30</td><td>30~40</td><td>40~50</td><td>50~60</td><td>> 60</td></tr>
<tr><td>Age (%)</td><td>7%</td><td>21%</td><td>44%</td><td>24%</td><td>3%</td></tr>
<tr><td></td><td>0~10</td><td>10~20</td><td>20~30</td><td>30~40</td><td>> 40</td></tr>
<tr><td>Professional seniority (%)</td><td>25%</td><td>32%</td><td>30%</td><td>10%</td><td>1%</td></tr>
<tr><td></td><td colspan="2">Rural</td><td colspan="2">Suburban</td><td>Urban</td></tr>
<tr><td>Urban context (%)</td><td colspan="2">29%</td><td colspan="2">14%</td><td>57%</td></tr>
<tr><td colspan="4">High-priority education network</td><td colspan="2">25%</td></tr>
<tr><td></td><td>Never</td><td>Rarely</td><td>3 to 4 times per month</td><td>1~2 times per week</td><td>3~4 times per week</td></tr>
<tr><td>Personal physical activity: frequency (%)</td><td>4%</td><td>18%</td><td>7%</td><td>47%</td><td>23%</td></tr>
<tr><td colspan="5">Sport license (club, association)</td><td>38%</td></tr>
</table>

Fig 1. University education of participating teachers.

Table 3. School context: number of classes, number of pupils per school and per class, number of pupils per cycle and per level (Cycle 1: TPS Toute Petite Section, MS Moyenne Section and GS Grande Section; Cycle 2: CP Cours Préparatoire, CE1 Cours Elémentaire 1 and CE2 Cours Elémentaire 2; Cycle 3: CM1 Cours Moyen 1 and CM2 Cours Moyen 2), single and multigrade class, sport school association, APQ implementation and external contractor.

Number of classes per school (percentage)	<5 classes	5~10 classes	10~15 classes	>15 classes
	43%	24%	23%	10%

Total number of pupils per school (percentage)	<50	50~100	100~200	200~300	300~400	>400
	7%	17%	40%	22%	10%	4%

Number of pupils per class (percentage)	<15	15~20	20~25	25~30	>30
	7%	11%	49%	31%	2%

Cycle	Cycle 1				Cycle 2		Cycle 3			Specific needs (6~11)
Class (pupils age)	TPS (2~3)	PS (3~4)	MS (4~5)	GS (5~6)	CP (6~7)	CE1 (7~8)	CE2 (8~9)	CM1 (9~10)	CM2 (10~11)	
Number of pupils per level (percentage)	2%	16%	9%	10%	10%	10%	12%	13%	18%	< 1%

Single-grade class			Multigrade class				
58%			42%				
Cycle 1	Cycle 2	Cycle 3	Cycle 1	Cycle 2	Cycle 3	Cycle 1/2	Cycle 2/3
18%	23%	17%	17%	8%	9%	2%	7%

Existence of a sport school association (‘USEP’)		13%
APQ implementation		55%
External contractor in PE	No	60%
	Yes	40%

Table 4. Mean (± standard deviation) durations of physical education, per department, for pre- and elementary school and per cycle.

Preschool (Cycle 1)	Nord (59)	2h21min ± 1h3min
	Pas-de-Calais (62)	2h23min ± 55min

	Seine-et-Marne (77)	2h20min ± 1h1min
	Vienne (86)	3h04min ± 1h5min
	Seine-Saint-Denis (93)	2h10min ± 1h7min
	Val-de-Marne (94)	2h20min ± 1h13min
Elementary school (Cycles 2 & 3)	Nord (59)	2h46min ± 48min
	Pas-de-Calais (62)	2h13min ± 41min
	Seine-et-Marne (77)	2h32min ± 54min
	Vienne (86)	2h34min ± 46min
	Seine-Saint-Denis (93)	2h10min ± 49min
	Val-de-Marne (94)	2h17min ± 56min

Fig 2. Primary obstacles reported by participating teachers.

Weekly PE durations (table 4) were respectively 2h23min ± 1h04min in preschool (cycle 1), and 2h28min ± 51min in elementary school (cycle 2: 2h25min ± 51min; cycle 3: 2h31min ± 51min), inferior to the respective mandatory weekly 2h30 and 3h ($p<0.01$). PE durations were also different between departments, both in pre- and in elementary school ($p<0.001$), with a higher value in preschool for Vienne ($p<0.001$ vs. other departments), and in elementary school for Vienne and Nord (no difference between them, $p=0.347$, and $p<0.01$ vs. other departments).

Peri-PE duration (data not shown) was impacted by cycle ($p<0.001$): peri-PE was lower in preschool (cycle 1) than in elementary school (cycle 2 and cycle 3, no difference between them, $p=0.991$).

PE durations and teacher's background

Men spend more time than women in PE teaching ($p=0.003$), more specifically in elementary ($p=0.026$) but not in pre-school ($p=0.138$).

There was an effect of age ($p=0.017$) and seniority ($p<0.001$) on PE duration. However, there was no effect of initial university cursus on PE durations ($p=0.115$).

Personal PA frequency impacted durations of PE teaching ($p=0.021$), especially between ‘Never’ and ‘3 to 4 times a week’ ($p=0.036$). More specifically, PA frequency had a more important effect in women ($p=0.009$) but not in men ($p=0.818$). In the same manner, there was a tight trend, yet not significative, of practising sport in a club or an association (sport license) on PE duration ($p=0.054$).

PE durations and socio-demographic context

PE durations was strongly impacted by urban context ($p<0.001$), lower in urban vs. suburban/rural environment ($p<0.001$ for both) but not different between rural and suburban ($p=0.618$). Inhabitant numbers also had an effect ($p<0.001$), especially for ‘2 000~5 000’, higher than ’15 000~30 000’ and ’30 000~60 000’ ($p=0.006$ and $p=0.007$, respectively).

Finally, PE durations was lower in high-priority areas (REP and REP+) than in other areas ($p<0.001$). More specifically, this difference was present in elementary school ($p=0.002$) whereas it was only a trend in pre-school ($p=0.087$).

PE durations and school context

Number of school classes impacted PE durations ($p<0.001$), in preschool and in elementary school ($p=0.044$ and $p<0.001$, respectively), especially with higher durations in smaller schools: ‘<5 classes’ vs. ’5~10 classes’ and ’10~15 classes’ ($p<0.001$ and $p=0.005$, respectively).

In the same manner, there was an effect of the total number of pupils on school PE durations ($p=0.007$), with, for example, a higher volume in the ‘100~150’ category than in the ‘300~350’ one ($p=0.012$). However, there was no effect of number of pupils in class on PE durations ($p=0.088$), except in elementary school ($p=0.022$), but not in preschool ($p=0.701$).

PE duration was longer in multigrade classes than in single-grade ones ($p<0.001$), both in pre- and elementary schools ($p<0.001$ and $p=0.012$, respectively).

The presence of a school sport association (USEP) also increases the PE duration ($p<0.001$), in elementary school ($p=0.007$) but not in preschool ($p=0.232$).

Similarly, the implementation of APQ also increases the PE duration ($p<0.001$), in elementary school ($p=0.001$) but not in preschool ($p=0.790$).

Overall, PE duration was longer with an external contractor than without one both in elementary school ($p<0.001$) and in preschool ($p=0.044$).

PE and obstacles

Primary obstacles are presented in figure 2.

The main primary obstacles, ranked first in PT answers, were, in order of importance: lack of time (32%), followed by adverse weather conditions (19%) and lack of infrastructures (17%). However, separating preschool teachers from elementary ones, primary obstacles were different: preschool teachers gave importance mostly to lack of time (21%), lack of infrastructures (17%), and other obstacles (25%) whereas elementary teachers placed in order lack of time (38%), weather (23%) and lack of infrastructures (18%) (not showed).

When obstacles are pooled together without ranking them (data not shown), the order of obstacles remains the same, with slightly different percentages: lack of time (23%), followed by adverse weather conditions (22%) and the lack of infrastructures (16%). In the same manner than in primary obstacles, preschool teachers placed most importance mostly to lack of time and lack of infrastructures (both 16%), followed by weather and other obstacles (both 15%), whereas elementary teachers listed first lack of time and weather (both 25 %), and lack of infrastructures (16%).

PE and professional skills

Overall, 22.4% of PT stated having no affinity for PE teaching (18.7% and 24.4% in pre- and in elementary school, respectively). Having a higher affinity for PE teaching augmented PE durations ($p=0.016$), which we observed in elementary school ($p<0.001$) but not in preschool ($p=0.745$).

There was also an effect of the feeling of competence on PE durations: the more competent the teacher felt, the longer PE durations were ($p<0.001$), except between ‘I do not feel competent’ and ‘I do not

feel very competent' (p=0.646), and between 'I feel competent enough' and 'I feel very competent' (p=0.542). More precisely, this effect was observed similarly in elementary teachers ($p<0.001$, with similar differences between intra-levels) and in pre-school teachers (p=0.028, with a sole difference between 'I do not feel very competent' and 'I feel competent enough', p=0.03).

An initial training in PE pedagogy during teaching formation augmented PE durations (data not shown), whether it was judged sufficient or not (p=0.040). However, continuous training in PE, during , did not modify PE duration ($p<0.774$).

Discussion

This study took place in several French departments (table 1) and the survey was based on teachers' free will answers, although French primary teachers endure high psychological and workload pressures, between teaching itself (including class management), session preparations, grading/proofreading, team working/meetings, and administrative duties [17], for lower salary and attractivity than in other European countries [18] . Considering this non-negligeable constraints, the apparent low overall answer rate for a non-mandatory task (below 4%, table 1) can nevertheless be recognized as a first positive highlight in this study.

As observed in previous works and government reports [14,15,19,20], our present work points out that both preschool and elementary teachers failed to meet the duration of program requirements in Physical Education (table 4). However, the observed difference against the official instructions was lower for pre-school (< 5%) than for elementary school (-18%), which might be due to the presence in most preschools of a fully dedicated space or room for PE, along with a specific motricity course to be used on a daily basis, at predetermined times, for long periods (6 to 12 weeks according to pedagogic curriculum), as per official program recommendations [13], whereas PE material in elementary school usually has to be reset up at each PE session. This pedagogic time-related efficiency in preschool schedules, here illustrated by a permanent material setup for PE, was

previously observed in France [15] and in USA [21]. We can also relate this to the peri-PE durations (transportation to gymnasium, clothes changing, …), which are lower in preschool than in elementary school: if the majority of PE sessions in preschool are performed every day at the same dedicated place inside the school, then these teachers save a non-negligible amount of time in transportation and setup and can relocate the "saved time" into PE session itself, e.g. "actual" physical activity.

Beyond this main result, further differences were found on PE durations regarding the distinct influences of the PT background and the school context. Considering individual characteristics, the first effect is linked to PT sex (table 1): male PT spend more time teaching PE, which was previously observed [15], especially in elementary school, whereas no difference between men and women was observed in preschool. The aforementioned presence of a dedicated and accessible setup for several weeks may smooth out the existing difference between men and women, and act as a facilitator for PE teaching. PE duration also augments with age and seniority (table 2): it is obvious that the subsequent gain in pedagogic experience coming from these two dimensions illustrates a better efficiency at organising and teaching PE, increasing over the years [15,22]. However, it is unexpected that the training in PE during the initial professional formation is a determinant factor for PE duration in practise afterwards, whereas continuous training does not have an effect on it. Literature is lacking on this matter: a sole French study showed a positive effect of the perceived quality (not spent time) of initial and continuous training in PE teaching [15] whereas an English one showed the opposite for both [23]; our findings lie between the two, and none of these studies focused on the content of initial and continuous training, which may play a key-role here.

Another important corollary is that the initial university cursus (fig 1) does not impact PE duration, contrary to other recent study [15]. One could hypothesize that having studied sport sciences could facilitate and encourage PE teaching, especially since the university curriculum of sports science includes some specific topics about PE teaching, but our data does not support this point: teachers with specific background skills in sports and physical activities do not meet the mandatory teaching

cursus in PE, just as teachers with different backgrounds. We can here conjecture that these teachers meet the same general obstacles than their colleagues that keep them from reaching duration objectives, despite their specific professional skills. In this matter, it is interesting to distinguish the effects of the background in sport studies from personal sport habits, in club or not: our data point out that the current relationship to sports and physical activities (table 2), e.g. personal sport habits, especially with female teacher (frequency, club license, table 2), weighs more on the time spent to teach PE than a specific initial training in the didactic field, as observed elsewhere [15].

Finally on the individual aspect, both affinity and feeling of competence for PE teaching impact PE duration. Being linked to age and seniority, the growing feeling of competence is associated with a greater time spent teaching PE: the more confident teachers are in their teaching skills, the more time they teach PE, which corroborates previous works [15,24]. Similarly, and beneficial for building the feeling of competence [25], the initial training in PE teaching (different from initial university cursus) also plays a role in PE teaching duration afterwards: both initial PE teaching training and "field experience" are potent factors for PE duration, in line with other studies focusing on the perceived quality of this initial training [15].

Beyond personal background and history, geographic, socio-economic and school contexts are of importance too. First, both urban context (table 2) and number of town inhabitant (data not presented) lower PE duration, which is different to the scarce data on this subject [26]. It is likely that rural surrounding provides more opportunities for outdoor PE, such as open-air activities (orienteering, kayaking, collective sports, …) than schools located in dense urban areas. Moreover, and paradoxically, the presence of external contractors (table 3), more available in urban areas than in rural ones (data not presented) [27], does not augment PE duration, which potentially implies that the mean PE duration would be lower without these external interventions. This raises a long-known issue about these interventions: while primary teachers are pedagogically accountable for applying the entirety of national program, PE included, they tend to delegate PE teaching to the external

contractor (usually a coach from a club or a sport association, or a town hall officer), leading to a full disciplinary content as teached in middle and high schools, contrary to guidelines of Ministry of Education [28]. Moreover, in REP/REP+ zones located in urban and suburban areas, a vast majority of primary teachers mention the additional difficulties of pupil management intrinsically linked to the low socioeconomic status of these areas [29], which are amplified outside the classroom, i.e. during PE. In a more school-centred topic, school class numbers, total number of pupils in school and per class lowered PE duration: whereas a larger class size does not seem to impact the global academic results, it certainly modifies the management behaviour by the teacher [30], especially by reducing grouping work opportunities beneficial for pupils with learning difficulties [31]; in PE, the intrinsic specificity of this subject (open-air, continuous mobility, material, loud environment, safety rules, …) can increase a reluctance in teachers to engage in its teaching [32]. Moreover, a greater number of classes in a school raises planning issues about sharing spaces already in limited numbers, which can discourage PT for PE teaching. On another side, unexpectedly, multigrade classes are spending more time in PE than single-grade ones (table 3), which is surprising due to further difficulties regarding the various curriculums, the necessary pedagogic differentiations and adaptations, and the management of pupils heterogeneity [33]. To our knowledge, there are no studies focused on the benefits of PE in multigrade class, but we can hypothesize that well-known global psychosocial effects of multigrade class in pupils can be applied to PE as well, such as autonomy, cooperation and socialisation [34]. The presence of a sport school association (USEP, table 3) also enhances PE duration, as USEP trainers/coaches are usually PT: as personal sport habits, it is legitimate to assume the existence of skills and affinity transfers from USEP to PE teaching, although there is currently not enough data in the present study to support this point. Experimented from 2020 and then prescribed by legislation in 2022 [35], APQ (‘Activités Physiques Quotidiennes’, Daily Physical Activity) was implemented in French primary school for public health purposes, in order to counteract deleterious effects of sedentary lifestyles. However, in accordance with official texts, APQ is distinct

from PE and represents a complement to the existing PE: although mandatory as per national program, our study shows that only 55% of primary teachers (table 3) effectively implement APQ in their planning. Our data is in line with previous publications: primary teachers may be reluctant to apply this imposed and ill-defined measure, and tend to merge both of them into a single subject, e.g. Physical Education [36]. We designed our questionnaire to clearly distinguish PE from APQ, as it was specified in French official program; however, a Senate report pointed out the confusion and the semantic difference between both, which led a non-negligible number of teachers to "artificially" increase PE duration by including APQ into PE [37]. This implies that the actual mean PE duration might be lower than the one we evaluated, although we do not have the means to verify this matter.

Regarding obstacles declared and faced by teachers for PE teaching (fig 2), most authors point out, among others, the lack of formation (initial and continuous), insufficient self-confidence, specific group management and lack of infrastructures and materials [15,20,23,32], which was confirmed by our data. However, according to the present study, the greatest obstacle was the lack of time (fig 2), for both pre- and elementary school. This reason was evoked, although not quantified, by large-scale studies in previous works and reports: teachers, under government pressure [20,38,39], prioritize French language and mathematics teaching over other subjects such as PE [14,40]. The second greatest obstacle, equally poorly documented, is weather conditions: we noted a difference in PE duration between departments (tables 1 and 4), Vienne and Nord being the ones with the highest; however, according to national public weather archives, neither number of sunny or rainy days does not seem to influence PE duration (data not presented), and further studies in other departments would be relevant to assess the effects of daily climate on PE duration.

Conclusion

Our present study confirms previous works: the current teaching of physical education does not meet the requirements of national program, and is mainly impacted by teachers' sex, age, seniority, PA

habits, urban and socio-economic contexts, the presence of an external contractor, pupil numbers (in elementary schools), affinity and feeling of competence.

We are aware, given that this survey was completed solely on voluntary will in a very tight time schedule, that maybe most answering teachers showed an interest in PE teaching, and a wider survey would be relevant to confirm, tune or infirm our findings.

Regarding public health, PE in primary schools is of crucial importance to prevent sedentary habits and its subsequent comorbidities (obesity, diabetes mellitus, cardiovascular pathologies, etc.): primary school represents the most relevant institution to identify such potential children and act accordingly [41].

This article only aims to describe, at a given time, the relationship between primary teachers and physical education, and what could be the factors weighing on it. We do not suggest any solution at this point, as potential propositions should be tested and analysed in further studies, knowing that numerous courses of actions on the field are plausible, going from engagement, motivation, initial and continuous training and pedagogic differentiation.

Acknowledgments

We are very grateful to academic authorities of each department for making this study possible.

References

1. Valenzuela PL, Carrera-Bastos P, Castillo-García A, Lieberman DE, Santos-Lozano A, Lucia A. Obesity and the risk of cardiometabolic diseases. Nat Rev Cardiol. 2023;20: 475–494. doi:10.1038/s41569-023-00847-5

2. Elagizi A, Kachur S, Carbone S, Lavie CJ, Blair SN. A Review of Obesity, Physical Activity, and Cardiovascular Disease. Curr Obes Rep. 2020;9: 571–581. doi:10.1007/s13679-020-00403-z

3. Lioret S, Campbell KJ, McNaughton SA, Cameron AJ, Salmon J, Abbott G, et al. Lifestyle Patterns Begin in Early Childhood, Persist and Are Socioeconomically Patterned, Confirming the Importance of Early Life Interventions. Nutrients. 2020;12: 724. doi:10.3390/nu12030724

4. Deschamps V, Salanave B, Torres M, Verdot C. Étude de santé sur l'environnement, la biosurveillance, l'activité physique et la nutrition (Esteban), 2014-2016. Volet Nutrition. Chapitre Activité physique et sédentarité. 2ème édition. Saint-Maurice: Santé publique France; 2020.

5. Vanhelst J, Lang JJ, Matelot D, Carré F, Mercier D, Ulmer Z, et al. Cardiorespiratory fitness has declined among French children since 1999, although the decline appears to be getting smaller. Scandinavian Med Sci Sports. 2024;34: e14641. doi:10.1111/sms.14641

6. Moore LL, Lombardi DA, White MJ, Campbell JL, Oliveria SA, Ellison RC. Influence of parents' physical activity levels on activity levels of young children. J Pediatr. 1991;118: 215–219. doi:10.1016/s0022-3476(05)80485-8

7. Trost SG, Sallis JF, Pate RR, Freedson PS, Taylor WC, Dowda M. Evaluating a model of parental influence on youth physical activity. Am J Prev Med. 2003;25: 277–282. doi:10.1016/s0749-3797(03)00217-4

8. Tudor-Locke C, Ainsworth BE, Popkin BM. Active commuting to school: an overlooked source of childrens' physical activity? Sports Med. 2001;31: 309–313. doi:10.2165/00007256-200131050-00001

9. Baranowski T, Thompson WO, DuRant RH, Baranowski J, Puhl J. Observations on physical activity in physical locations: age, gender, ethnicity, and month effects. Res Q Exerc Sport. 1993;64: 127–133. doi:10.1080/02701367.1993.10608789

10. ONAPS. Activité physique et sédentarité de l'enfant et de l'adolescent. Nouvel état des lieux en France. Paris: ONAPS; 2022.

11. Bueno-Antequera J, Munguía-Izquierdo D. Physical Inactivity, Sedentarism, and Low Fitness: A Worldwide Pandemic for Public Health. In: Rezaei N, editor. Integrated Science of Global Epidemics. Cham: Springer International Publishing; 2023. pp. 429–447. doi:10.1007/978-3-031-17778-1_19

12. Guinhouya BC, Lemdani M, Apété GK, Durocher A, Vilhelm C, Hubert H. How School Time Physical Activity Is the "Big One" for Daily Activity Among Schoolchildren: A Semi-Experimental Approach. Journal of Physical Activity and Health. 2009;6: 510–519. doi:10.1123/jpah.6.4.510

13. Ministère de l'Education Nationale de la Jeunesse et des Sports. Bulletin officiel n° 25 du 24 juin 2021. 2021. Available: https://www.education.gouv.fr/bo/21/Hebdo25/MENE2116550A.htm

14. Loarer C, Sallé J. La pratique sportive à l'école primaire. Inspection Générale de l'Education Nationale; 2012 May. Available: https://www.education.gouv.fr/cid60660/la-pratique-sportive-a-l-ecole-primaire.html

15. Escriva-Boulley G, Tessier D, Sarrazin P. Augmenter le temps d'enseignement de l'éducation physique et sportive à l'école primaire : sur quels facteurs peut-on agir ? Revue française de pédagogie Recherches en éducation. 2018; 33–46. doi:10.4000/rfp.8406

16. World Medical Association. World Medical Association Declaration of Helsinki: Ethical Principles for Medical Research Involving Human Participants. JAMA. 2025;333: 71. doi:10.1001/jama.2024.21972

17. Laugaa D, Rascle N, Bruchon-Schweitzer M. Stress and burnout among French elementary school teachers: A transactional approach. European Review of Applied Psychology. 2008;58: 241–251. doi:10.1016/j.erap.2008.09.007

18. OECD. Education at a glance 2024: OECD indicators. Paris: OECD Publishing; 2024.

19. Cour des Comptes. L'école et le sport : une ambition à concrétiser. Cour des Comptes; 2019 Sept. Available: https://www.ccomptes.fr/system/files/2019-09/20190912-rapport-ecole-et-sport.pdf

20. Claus P, Bouysse V, Mégard M, Auverlot D, Durpaire J-L, Jardin P, et al. Bilan de la mise en œuvre des programmes issus de la réforme de l'école primaire de 2008. Paris: Inspection Générale de l'Education Nationale; 2013. Report No.: 2013–066.

21. Perna FM, Oh A, Chriqui JF, Mâsse LC, Atienza AA, Nebeling L, et al. The Association of State Law to Physical Education Time Allocation in US Public Schools. Am J Public Health. 2012;102: 1594–1599. doi:10.2105/AJPH.2011.300587

22. Schempp PG, Manross D, Tan SKS, Fincher MD. Subject Expertise and Teachers' Knowledge. Journal of Teaching in Physical Education. 1998;17: 342–356. doi:10.1123/jtpe.17.3.342

23. Harris J, Cale L, Musson H. The predicament of primary physical education: a consequence of "insufficient" ITT and "ineffective" CPD? 2012 [cited 1 July 2025]. doi:10.1080/17408989.2011.582489']

24. Faulkner G, Reeves C, Chedzoy S. Nonspecialist Preservice Primary-School Teachers: Predicting Intent to Teach Physical Education. Journal of Teaching in Physical Education. 2004;23: 200–215. doi:10.1123/jtpe.23.3.200

25. Borko H, Putnam RT. Learning to teach. Handbook of educational psychology. London, England: Prentice Hall International; 1996. pp. 673–708.

26. Pfledderer CD, Burns RD, Byun W, Carson RL, Welk GJ, Brusseau TA. School-based physical activity interventions in rural and urban/suburban communities: A systematic review and meta-analysis. Obesity Reviews. 2021;22: e13265. doi:10.1111/obr.13265

27. Querio T. Pluralité de l'éducation physique et sportive dans le premier degré et étude de ses déterminants. Un enjeu de démocratisation scolaire ? Université de Rennes 2. 2019. Available: https://dumas.ccsd.cnrs.fr/dumas-02311228

28. Amans-Passaga C. Analyse didactique de situations de partenariats professeur / intervenant, en EPS à l'école primaire: Recherches en didactiques. 2013;16: 43–58. doi:10.3917/rdid.016.0043

29. Foy L. Des pratiques inavouables : la gestion du désordre scolaire dans les écoles de la grande pauvreté. champpenal. 2023;29. doi:10.4000/champpenal.14625

30. Blatchford P, Russell A. Class size, grouping practices and classroom management. International Journal of Educational Research. 2019;96: 154–163.

31. Spear-Swerling L. Structured Literacy and Typical Literacy Practices: Understanding Differences to Create Instructional Opportunities. TEACHING Exceptional Children. 2019;51: 201–211. doi:10.1177/0040059917750160

32. Durand C, Richard D. Enseigner l'EPS à l'école primaire. 2e édition. Malakoff: Dunod; 2020.

33. Acatrinei IR, Popovici AO. The organization of multigrade teaching in primary school. Challenges and chances. ICERI2021 Proceedings. 2021; 1627–1633. doi:10.21125/iceri.2021.0442

34. Little A. Education for all and multigrade teaching: challenges and opportunities. Dordrecht, The Netherlands: Springer; 2006.

35. Loi n° 2022-296 visant à démocratiser le sport en France. 2022-296 Mar 2, 2022. Available: https://www.legifrance.gouv.fr/eli/loi/2022/3/2/MENX2105502L/jo/texte

36. Hermand E, Rouffet L. Activité physique et cognition, clefs de la réussite à l'école primaire. Revue EP&S. 2023; 34–37.

37. Gosselin B, Darcos L. Redonner du souffle aux "30 minutes d'activité physique quotidienne à l'école" pour améliorer la santé des élèves. Paris: Sénat; 2024. Report No.: 774.

38. Mégard M, Bouysse V, Debuchy V, Lepetit M-L, Loarer C, Rimane J. Efficacité pédagogique de la réforme des rythmes scolaires. Inspection Générale de l'Education Nationale; 2015 June. Report No.: 2015–042. Available: https://www.vie-publique.fr/rapport/36033-efficacite-pedagogique-de-la-reforme-des-rythmes-scolaires

39. Pontais C, Thépaut A, Verscheure I. L'enquête des « écoles vitaminées » à l'EPS et perspectives de recherche. eJRIEPS Ejournal de la recherche sur l'intervention en éducation physique et sport. 2024 [cited 1 July 2025]. doi:10.4000/11qez

40. Blanchouin A. La dynamique d'une journée de classe au CP. ContrePied. 2016; 28–29.

41. Ortega FB, Ruiz JR, Castillo MJ, Sjöström M. Physical fitness in childhood and adolescence: a powerful marker of health. Int J Obes. 2008;32: 1–11. doi:10.1038/sj.ijo.0803774

Figure 1

Click here to access/download;Figure;Fig1.tif

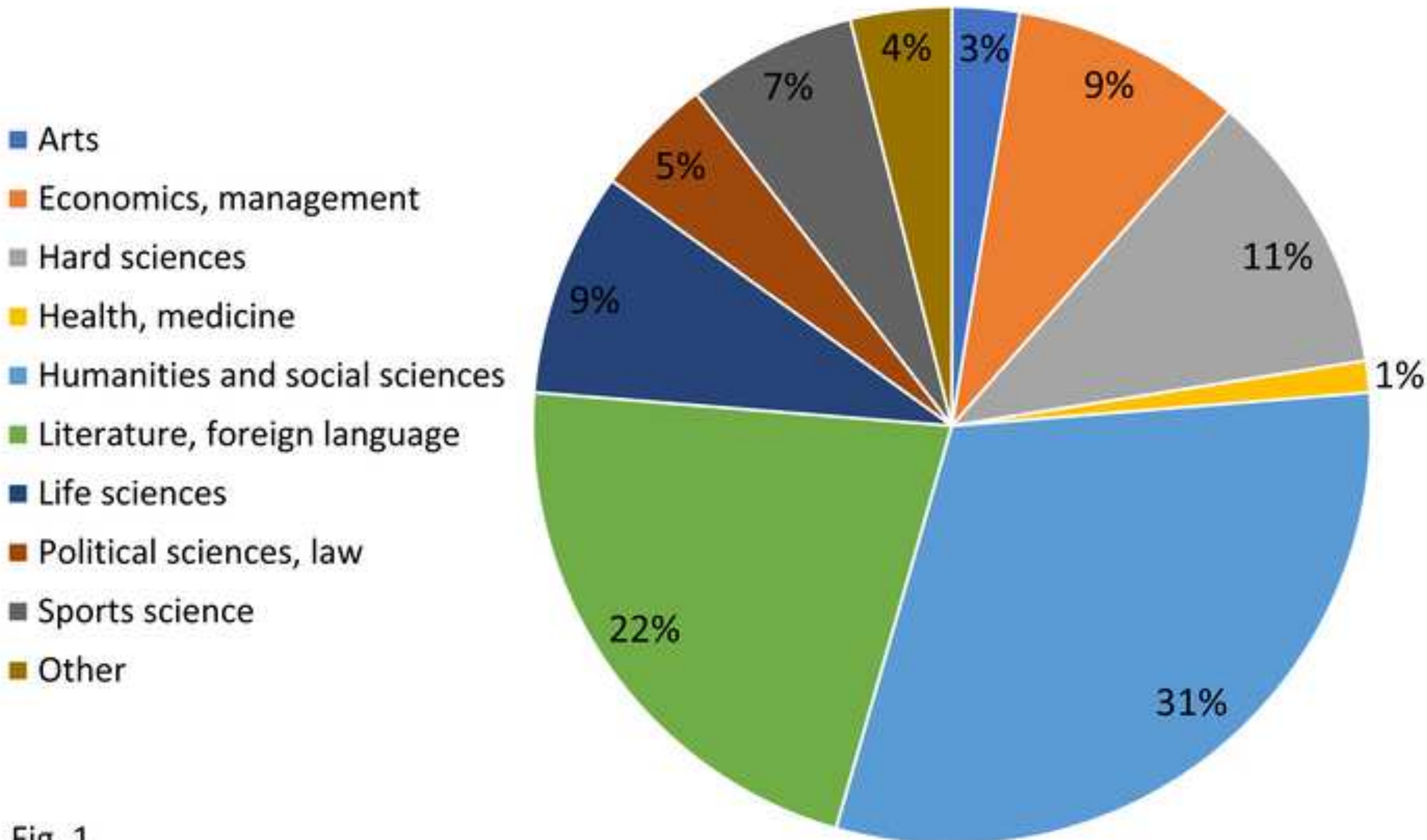

Fig. 1

Figure 2

Click here to access/download;Figure;Fig2.tif

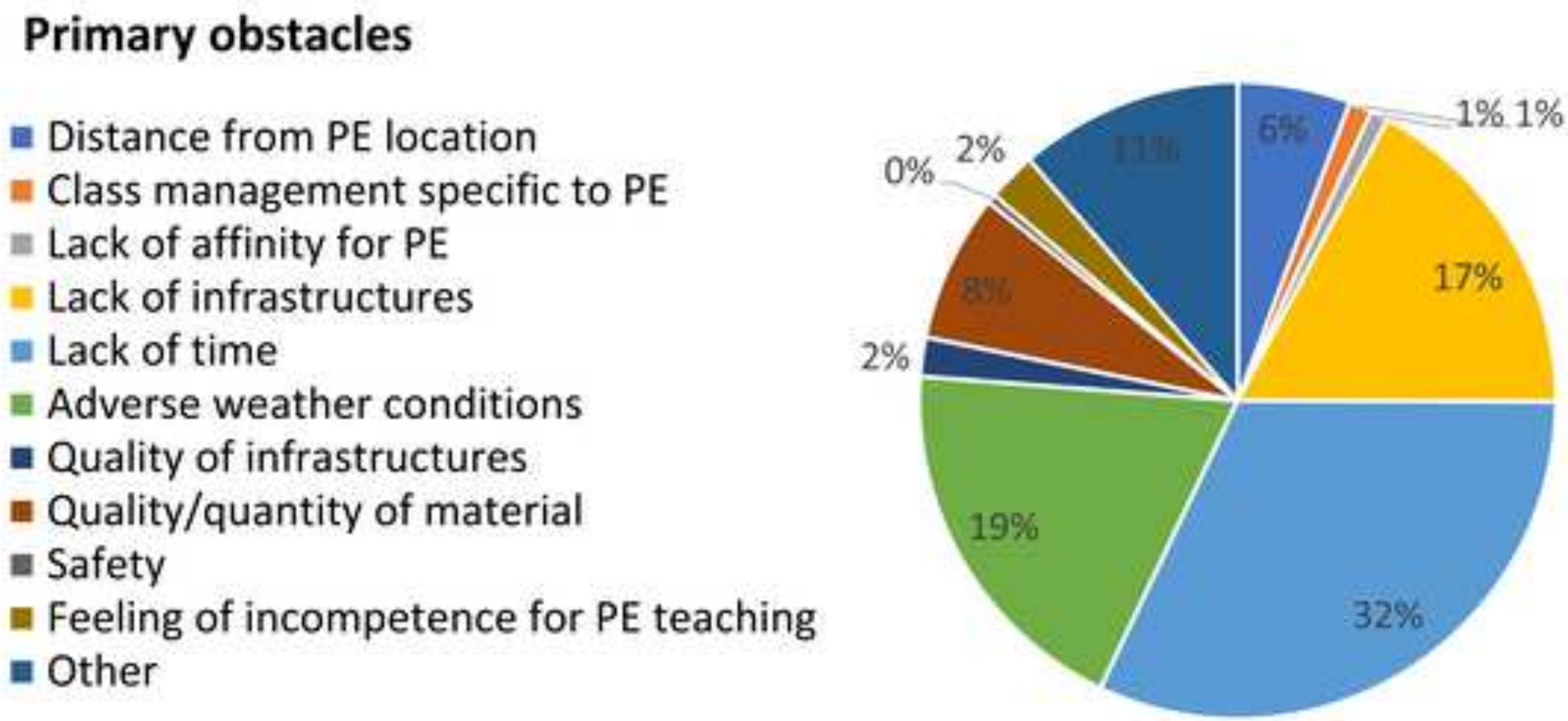

Fig. 2
