# Supplementary material for: Duration and barriers of physical education teaching in primary schools in 6 French departments
Source: PLoS One. 2026 Nov 3;21(11):e0362195. doi: 10.1371/journal.pone.0362195 (PMC13730362; doi:10.1371/journal.pone.0362195)
Supplement: Supplementary file 2 [file pone.0362195.s002.docx]

October 10^th^, 2024

**Ref # PONE-D-26-17765**

**“Physical Education in Primary schools: an overview of teaching practices in France”**

**_________________________________________________________________________________**

Dear Editor,

Thank you for your email dated June 5^th^, 2024 regarding our manuscript “Physical Education in Primary schools: an overview of teaching practices in France”.

First, we would like to thank the reviewers for the time and efforts in evaluating our manuscript and for their valuable comments. Second, thanks to their remarks and comments, we deeply modified our manuscript, especially regarding the statistical methodology (multivariable regression model), which brings a more accurate analyses about factors influencing the duration of physical education in primary school. We also replied point by point to the additional comments and revised the manuscript accordingly. You will find the answers to the reviewers’ comments below.

We hope this revised manuscript is now suitable for publication in your journal. If you have additional questions or comments, please do not hesitate to contact us.

Sincerely yours,

Eric Hermand

**Editorial comment :**

1. Please ensure that your manuscript meets PLOS ONE's style requirements, including those for file naming. The PLOS ONE style templates can be found at

<https://journals.plos.org/plosone/s/file?id=wjVg/PLOSOne_formatting_sample_main_body.pdf> and

<https://journals.plos.org/plosone/s/file?id=ba62/PLOSOne_formatting_sample_title_authors_affiliations.pdf> .

**Author’s note :** yes, we took the PLOS One formatting into account.

2. Thank you for submitting to PLOS One. We note that you describe receiving explicit, informed consent from participants. Before we can proceed, please specify how consent was witnessed or documented in the questionnaire presentation. For example, was consent written, verbal, did participants have to answer a specific question in the questionnaire granting consent?

**Author’s note :** we clarified this point in the methodology section.

3. In the online submission form, you indicated that “This study was performed under the supervision and agreement of Academic departmental authorities. The data that support the findings of this study are available from the corresponding author, Dr. Eric Hermand, upon reasonable request”.

All PLOS journals now require all data underlying the findings described in their manuscript to be freely available to other researchers, either a. In a public repository, b. Within the manuscript itself, or c. Uploaded as supplementary information. This policy applies to all data except where public deposition would breach compliance with the protocol approved by your research ethics board. If your data cannot be made publicly available for ethical or legal reasons (e.g., public availability would compromise patient privacy), please explain your reasons on resubmission and your exemption request will be escalated for approval.

**Author’s note :** after discussions with concerned parties, I will uploaded the necessary data for analyses and interpretation, as a supplementary information.

4. We note that you have included the phrase “data not shown” in your manuscript. Unfortunately, this does not meet our data sharing requirements. PLOS does not permit references to inaccessible data. We require that authors provide all relevant data within the paper, Supporting Information files, or in an acceptable, public repository. Please add a citation to support this phrase or upload the data that corresponds with these findings to a stable repository (such as Figshare or Dryad) and provide and URLs, DOIs, or accession numbers that may be used to access these data. Or, if the data are not a core part of the research being presented in your study, we ask that you remove the phrase that refers to these data.

**Author’s note :** in addition to data availability in supplementary information and modification of statistical methodology (as required by reviewers), we only kept necessary data to our analyses and further removed unnecessary ones.

5. Please include your full ethics statement in the ‘Methods’ section of your manuscript file. In your statement, please include the full name of the IRB or ethics committee who approved or waived your study, as well as whether or not you obtained informed written or verbal consent. If consent was waived for your study, please include this information in your statement as well.

**Author’s note :** we clarified this point : as this questionnaire was completely anonymous, did not provide any information allowing to identify any participant, and did not require participants to disclose any racial, ethnic, politic, religious, sexual, health, biometric and genetic information, an ethics committee approval was NOT required.

6. If the reviewer comments include a recommendation to cite specific previously published works, please review and evaluate these publications to determine whether they are relevant and should be cited. There is no requirement to cite these works unless the editor has indicated otherwise.

**Author’s note :** the reviewers provided useful suggestions and did not require to cite any specific articles. We greatly appreciate that.

**Reviewer #1 comments**

The manuscript addresses an important topic and benefits from a large sample and its focus on PE duration and barriers in French primary schools. However, several revisions are needed before publication.

1. The title should better reflect the main focus on PE duration and related barriers.

**Author’s note :** thanks for this suggestion, we modified the title accordingly.

2. The abstract should mention the response rate and its implications for representativeness.

**Author’s note :** we added this information in the abstract.

3. The statistical analysis should be strengthened by reporting effect sizes, confidence intervals, adjustment for multiple comparisons, and, preferably, multivariable models.

**Author’s note :** thank you for this suggestion, which is in line with other reviewers’ ones. We completely redesigned the statistical analyses, by including a multivariable regressive model. This allowed us to highlight the best predictors impacting PE duration.

4. The discussion should avoid causal language because the study is cross-sectional and self-reported, and the limitations should more fully address low response rate, selection bias, self-report bias, and lack of objective PE-duration verification.

**Author’s note :** indeed. We added these limitations at the end of the discussion.

5. Finally, the manuscript requires English language editing to correct grammatical and typographical errors.

**Author’s note :** this issue was addressed. We also thank reviewers for syntax and grammar suggestions.

**Reviewer #2 comments**

The manuscript addresses a relevant topic for education, public health, and school organization. The absolute sample size is substantial, with 1,967 responses from primary teachers across six French departments, and the study provides potentially useful descriptive information on reported physical education duration and perceived barriers to PE teaching. However, several methodological, statistical, and reporting issues currently limit the reliability and interpretability of the findings.

1. Data availability. The current data availability statement indicates that the data are available from the corresponding author upon reasonable request. This does not meet the expected standard for PLOS ONE unless clear legal or ethical restrictions prevent public sharing. The authors should deposit the minimal anonymized dataset and, where applicable, the analysis code in a public repository. If public sharing is not possible, the authors should clearly explain the specific restriction, who controls access, how access can be requested, and under what criteria data access would be granted.

**Author’s note :** after discussion with collaborators, we will provide the data.

2. Ethics approval and informed consent. The ethics statement reports approval by departmental authorities of the French Ministry of Education. However, it is unclear whether these authorities acted as an ethics committee or whether the study received a formal ethics waiver. Since the study involved human participants, even if adult teachers and anonymous responses were used, the authors should clarify the applicable ethical framework, the approving body, whether an approval or waiver number exists, and the exact form of informed consent obtained.

**Author’s note :** indeed, we were not clear enough about the distinction between the approval and the ethics approvals. Briefly, we needed the approval from departmental authorities to pass the questionnaire on, but no ethics approval was needed because the questionnaire was strictly anonymous and did not ask any questions about racial, ethnic, politic, religious, sexual, health, biometric and genetic background,

3. Sampling strategy and response rate. The sampling procedure needs further clarification. The manuscript states that teachers from 6,198 schools were contacted, but it is unclear how many individual teachers actually received the invitation. The response rate appears very low, around 3.9 percent overall, with important differences across departments. This raises a serious risk of non-response bias, especially because teachers with a greater interest in PE may have been more likely to participate. The authors should provide the true denominator, explain the recruitment procedure in detail, and, if possible, compare the sample with the target population in terms of sex, age, department, school type, and socioeconomic context.

**Author’s note :** we added a ‘Limitations’ about these matters and detailed some aspects in the methodology section.

4. Questionnaire development and validation. The manuscript describes the broad domains covered by the survey, but the questionnaire itself is not provided. The authors should include the complete questionnaire as supplementary material, including the exact wording of items, response options, and any branching logic. They should also describe how the questionnaire was developed, whether it was piloted, whether content validity was assessed, and whether any reliability checks were performed.

**Author’s note :** we will provide a questionnaire copy as supplementary material (in French).

5. Main outcome based on self-report. The main outcome, weekly PE duration, is based on teacher self-report. The authors should clarify whether respondents reported a typical week, a planned week, an average across the school year, or another timeframe. It is also important to explain how PE was distinguished from APQ and from peri-PE time. Given the possibility of recall bias and social desirability bias, the limitations of this measure should be discussed more explicitly.

**Author’s note :** thanks for these remarks, the were addressed in the methodology section and in the limitations section.

6. Data cleaning and exclusions. The Results section states that responses were retained after cleaning outliers, input errors, and duplicates. However, the manuscript does not explain how many responses were excluded, what criteria were used, or how duplicates were identified in an anonymous survey. The authors should provide a transparent flow diagram showing invited participants, responses received, exclusions, and final analytical sample.

**Author’s note :** these details were clarified in the results and limitations sections.

7. Statistical analysis. The statistical analysis is insufficiently described. The authors mention Shapiro-Wilk, Chi-square, Spearman, and Kruskal-Wallis tests, but they do not specify which test was used for each analysis, how post hoc comparisons were conducted, or how multiple comparisons were handled. The manuscript reports many p values, but does not provide effect sizes or confidence intervals. The authors should provide a more detailed statistical analysis plan, including treatment of missing data, assumptions, post hoc procedures, and correction for multiple testing where appropriate.

Need for multivariable analyses. Many of the variables examined are likely interrelated, including teacher age, seniority, sex, urban context, priority education network, school size, department, APQ implementation, USEP presence, and use of external contractors. The current analysis appears mostly bivariate. This limits the interpretation of the reported associations. The authors should consider multivariable models to identify factors independently associated with PE duration. If the structure of the data allows it, department or school-level clustering should also be considered.

**Author’s note :** thank you for these suggestions, which were also requested by other reviewers. We deeply modified the statistical analyses and switched to a multivariable analysis, which tuned (and modified) the results. This clearly enhanced the results analysis (despite the extra work !), thanks a lot for this.

9. Reporting of descriptive statistics. Table 4 reports mean PE durations by department, but it does not include the number of respondents per subgroup, confidence intervals, medians, or interquartile ranges. Since duration variables may not be normally distributed, robust descriptive statistics would be useful. The authors should provide n values for all subgroup analyses and consider presenting medians and interquartile ranges in addition to means and standard deviations.

**Author’s note :** tables and results section were modified accordingly.

10. Results reported as “data not shown”. Several important results are interpreted but not shown, including peri-PE duration, some APQ-related analyses, and other subgroup comparisons. Results that support the main claims should be available in the manuscript, supplementary tables, or the public dataset. The authors should avoid relying on “data not shown” for relevant conclusions.

**Author’s note :** indeed. These issues were addressed.

11. Interpretation of associations. The study design is cross-sectional and observational. Therefore, causal language should be avoided. Several sentences use language such as “impacted”, “increases”, or “lowered”, which may imply causality. The authors should use associative language throughout, such as “was associated with”.

**Author’s note :** thank you for the suggestion, we amended our manuscript accordingly.

12. Discussion and strength of conclusions. Some interpretations in the Discussion are plausible but not fully supported by the analyses presented. For example, explanations related to rural context, external contractors, multigrade classes, and USEP transfer effects require either additional data or more cautious wording. The conclusion should also be moderated because of the low response rate, voluntary participation, and self-reported nature of the data.

**Author’s note :** the modified statistical analysis provided some new interpretations. We used a more cautious wording.

13. Figures. Figures 1 and 2 are pie charts, which are not ideal for comparing multiple categories. Bar charts would improve readability. Figure 2 in particular should be presented as a horizontal bar chart with percentages and n values, and preferably stratified by preschool and elementary school if those differences are discussed in the text.

**Author’s note :** we first tried bar charts, but they were not very easy to read. Pie charts were finally the best solution for these figures, illustrated with percentages. The multivariable analysis did not point out pre- and elementary schools as significant factors, so there was no need for stratifications.

14. Language and formatting. The manuscript requires substantial English editing. Examples include “questionaries”, “this areas”, “personal et professional”, “teachers’age”, “significative”, “during ,”, “teached”, “non-negligeable”, and “in the same manner than”. The authors should also correct typographical and formatting issues, including the misspelling of Kruskal-Wallis and the apparent error in the reference list for reference 23.

**Author’s note :** these issues were addressed, thank you for pointing them out.

**Reviewer #3 comments**

The manuscript addresses an important topic and benefits from a large sample and its focus on PE duration and barriers in French primary schools. However, several revisions are needed before publication.

1. Abstract: The health rationale is clear, but the causal wording is too strong when it states that physical activity is the primary defence against sedentary lifestyles and chronic disease. Please soften this claim and state that regular physical activity is an important protective factor against health risks linked to sedentary behaviour.

**Author’s note :** these sentences were modified, thanks for pointing them out.

2. Introduction: The rationale is relevant, but the research gap needs sharper framing. The manuscript moves from child physical activity to PE teaching duration, yet it does not clearly explain why the selected departments can support broader conclusions about France. Please add a short paragraph that defines the gap, the novelty of this survey, and the limits of the geographic scope.

**Author’s note :** we clarified this point in the section leading to the aim and objectives of this study.

3. Materials and methods: The sampling strategy is a major weakness. The response rate is below 4%, and participation relied on voluntary answers, which creates a high risk of selection bias. Please explain how non-response bias was assessed or discuss more clearly that teachers interested in PE may be overrepresented.

**Author’s note :** these points were discussed the added limitation section.

4. The ethics statement needs more precision. The manuscript mentions approval by departmental authorities, but it does not give approval numbers or name a formal ethics committee. Please clarify whether formal ethics review was required, waived, or unavailable, and add the exact approval details where possible.

**Author’s note :** indeed, this was clarified in the methodology section. As this questionnaire was completely anonymous, did not provide any information allowing to identify any participant, and did not require participants to disclose any racial, ethnic, politic, religious, sexual, health, biometric and genetic information, an ethics committee approval was NOT required.

5. The questionnaire is not described in enough detail. The reader cannot assess item wording, validation, piloting, or reliability. Please add the questionnaire as supplementary material and describe how it was developed, tested, and distributed.

**Author’s note :** the questionnaire (in French) was added in the supplemental material.

5. Statistical analyses: The analysis reports many separate tests across many variables, but it does not mention correction for multiple comparisons or a multivariable model. This raises the risk of false-positive findings. Please consider adding a multivariable regression model and clarify whether p-values were adjusted.

**Author’s note :** thank you for these suggestions, which were also requested by other reviewers. We deeply modified the statistical analyses and switched to a multivariable analysis, which tuned (and modified) the results. This clearly enhanced the results analysis (despite the extra work !), thanks a lot for this.

6. Results: Some results are difficult to verify because several data are mentioned as “not shown.” This weakens transparency, especially for peri-PE duration and pooled obstacles. Please include these data in a table or supplementary file so readers can evaluate the full evidence.

**Author’s note :** these issues were fixed throughout the manuscript.

7. Table and figure presentation needs improvement. Figure 1 appears visually cropped, and Figure 2 is hard to read because part of the pie chart and labels are cut off. Please replace both figures with complete, high-resolution versions and consider bar charts instead of pie charts for clearer comparison.

**Author’s note :** I uploaded high-resolution figures (6000x4500 pixels, 600 ppp). I guess the PDF conversion executed by the website was not perfect. We first tried bar charts, but they were not very easy to read. Pie charts were finally the best solution for these figures, illustrated with percentages.

8. Discussion: The discussion often interprets associations as if they explain causes. For example, the manuscript suggests reasons for differences by sex, urban context, school size, and external contractors, but the cross-sectional design cannot confirm these mechanisms. Please rephrase causal explanations as hypotheses and keep conclusions aligned with the study design.

**Author’s note :** the entire discussion was rewritten in the light of the modified statistical analyses (multivariable model)

9. Several important confounders remain insufficiently controlled. Department, school size, urban context, priority education status, teacher profile, external contractor presence, and PE affinity may overlap. Please use an adjusted analysis to identify which factors remain associated with PE duration after controlling for the others.

**Author’s note :** We completely redesigned the statistical analyses, by including a multivariable regressive model. This allowed us to highlight the best predictors impacting PE duration.

10. Conclusion: The conclusion states that PE duration is mainly impacted by several factors, but the evidence is observational and self-reported. Please use more cautious wording, such as “was associated with,” and emphasize the descriptive nature of the study.

**Author’s note :** indeed. We rephrased the concerned sentences.

11. Data availability: The statement says that data are available from the corresponding author upon reasonable request, but PLOS usually expects the minimal dataset to be publicly available unless legal or ethical restrictions apply. Please deposit the anonymized dataset and code in a public repository, or clearly justify any restriction.

**Author’s note :** after discussions with concerned parties, I will uploaded the necessary data for analyses and interpretation, as a supplementary information

12. Language and style: The manuscript contains several grammar and wording problems, such as “questionaries,” “appetence,” “significative,” “teached,” “cursus,” and “teachers’age.” Please revise the manuscript with a fluent scientific English editor to improve clarity and consistency.

**Author’s note :** English language was corrected throughout the manuscript.

The main problems concern representativeness, transparency of the questionnaire and data, statistical control of multiple associations, and overinterpretation of cross-sectional findings. Please strengthen these points before the manuscript can support its claims with enough confidence.
