# Supplementary material for: Duration and barriers of physical education teaching in primary schools in 6 French departments
Source: PLoS One. 2026 Nov 3;21(11):e0362195. doi: 10.1371/journal.pone.0362195 (PMC13730362; doi:10.1371/journal.pone.0362195)
Supplement: Supplementary file 3 [file pone.0362195.s003.docx]

**Ref # PONE-D-26-17765**

**“Duration and barriers of physical education teaching in French primary schools”**

**_________________________________________________________________________________**

**Editorial comment :**

- Provide complete reporting of the multivariable model (coeﬃcients, 95% CIs, and model ﬁt), and correct the remaining statistical inconsistencies.

**Author’s note :** the Methods section was completed and a table was added.

- Clarify the criteria and numbers for each exclusion during data cleaning.

**Author’s note :** this was clarified in the Results section.

- Specify clearly how informed consent was documented.

**Author’s note :** the ethic section was modified et detailed as per requested.

- Revise title, abstract, and wording to reﬂect the geographical scope, self-reported nature of the data, and avoid causal language.

**Author’s note :** Done.

- Expand methodological details on sampling, response rate calculation, and potential selection bias.

**Author’s note :** the Methods section was modified accordingly.

- Correct the inconsistency regarding external contractors in Results vs. Discussion.

**Author’s note :** that issue was addressed.

**Reviewer #2 comments**

The revised manuscript has improved substantially and most of the previous concerns have been adequately addressed. I have only a few remaining points before publication:

- statistical reporting (pp. 16–20). Please provide a clearer and more complete report of the multivariable model, including the coeﬃcients and 95% CIs for the retained predictors and an overall measure of model ﬁt. There are also some apparent inconsistencies that should be checked: ten predictors are mentioned but only nine appear to be listed, and the reported partial f² value is negative.

**Author’s note :** the Methods section was completed and a table was added. The issue about predictors was addressed.

- Results (p. 20). Please correct the remaining statistical/wording inconsistencies. for example, “p>0.027” appears inconsistent with the statement that this comparison was signiﬁcant. Also, terms such as “inﬂuencing,” “augments,” “increase,” and “impact” should be replaced by associative wording given the cross-sectional design.

**Author’s note :** these issues in the Results section were adressed accordingly.

- Data cleaning (p. 17). The manuscript states that 73 responses were excluded because of input errors, duplicates, and aberrant answers. Please brieﬂy specify the number and criteria for each type of exclusion.

**Author’s note :** this was detailed at the beginning of the results section.

- Consent (pp. 14–15). Please clarify the exact mechanism by which informed consent was documented (e.g., explicit agreement/checkbox before accessing the questionnaire). the current wording appears to describe consent through participation/non-opposition rather than clearly stating how consent was recorded.

**Author’s note :** the ethic section was modified et detailed as per requested.

- Language. A ﬁnal English-language proofreading is still needed. several grammatical and wording issues remain throughout the Abstract, Methods, and Results.

**Author’s note :** the manuscript was proof-read accordingly.

Overall, these are relatively minor issues and the revised manuscript is much stronger.

**Author’s note :** we sincerely thank the reviewer for his/her help in that matter.

**Reviewer #3 comments**

I am glad to review your work, and I thank you for the opportunity to read and evaluate your manuscript.

The title currently suggests that the study provides an overview of Physical Education teaching practices in France, while the survey was conducted in only six French departments and the participation rate was very low. This wording may therefore imply a level of national representativeness that the study design does not support. Please consider specifying the geographical scope in the title, for example by referring to “six French departments,” so that the title accurately reflects the population investigated.

**Author’s note :** we modified the title following reviewers’ request.

In the Abstract, the main findings are clearly presented, but the study is described without mentioning that PE duration and barriers were self-reported by teachers and that the sample represented only a small proportion of the eligible population. These limitations are important for interpreting the results. Please consider briefly identifying the study as a questionnaire-based survey conducted in six departments and avoiding wording that may imply that the reported values represent all French primary schools.

**Author’s note :** we modified the abstract accordingly.

In the Introduction, the rationale for studying PE duration is relevant, but several statements concerning physical inactivity, sedentary behaviour, obesity, and the protective role of physical activity are presented in rather causal or absolute terms. These concepts are related but not interchangeable, and obesity is influenced by multiple biological, behavioural, social, and environmental factors. Please consider revising these statements so that the background reflects the multifactorial nature of obesity and clearly distinguishes physical inactivity from sedentary behaviour.

**Author’s note :** the introduction was modified accordingly, one reference was added.

In the Materials and Methods section, the sampling strategy and the calculation of the response rate require substantial clarification. You state that teachers from 6,198 primary schools were contacted through school principals and educational authorities, while the reported overall participation proportion is 3.9%. It is unclear whether this percentage was calculated using the total number of teachers in these departments, the number of teachers who actually received the invitation, or another denominator. Please provide the exact number of eligible teachers, the number who received the questionnaire, and the number who responded in each department, and distinguish clearly between a participation proportion and a true response rate.

**Author’s note :** details were added in the Methods, Results and Limitations sections.

The very low participation proportion also creates an important risk of nonresponse and self- selection bias. Teachers who chose to complete a voluntary questionnaire about PE may have had greater interest in physical activity or PE teaching than teachers who did not participate. This issue limits the generalizability of the findings considerably. Please compare the characteristics of your sample, where possible, with available characteristics of the overall primary teacher population and discuss more explicitly how selection bias may have influenced PE duration, affinity, competence, and physical activity measures.

**Author’s note :** these points were detailed and explained in the Methods section.

The data collection period extended from December 2021 to June 2024, which is particularly important because the educational context changed during this interval. For example, the manuscript states that the Daily Physical Activity initiative, APQ, became prescribed by legislation in 2022. Pooling responses obtained before and after such a policy change may introduce a temporal bias into the analysis. Please clarify when responses were collected within each year and consider analysing or adjusting the results according to the period of data collection, particularly for APQ-related outcomes.

**Author’s note :** these points were also covered in the discussion.

The Survey Design section describes the domains covered by the questionnaire but provides no information about questionnaire development, pilot testing, content validity, reliability, or previous validation. Since most of the conclusions depend directly on self-reported questionnaire data, the measurement properties of the instrument are essential for assessing the reliability of the findings. Please provide the complete questionnaire as supplementary material and explain how the items were developed, whether they were pilot-tested, whether previously validated questions were used, and how the response categories were selected.

**Author’s note :** the questionnaire was added in the supplementary data.

The description of the participants should also be revised because the manuscript initially refers to them as “PE teachers,” although the study appears to concern generalist primary school teachers who are responsible for teaching PE as part of the curriculum. This terminology may cause confusion, particularly because external PE contractors are analysed separately later in the manuscript. Please use a consistent term such as “primary school teachers” throughout the manuscript and reserve “PE teacher” for professionally specialised PE teachers if such participants were actually included.

**Author’s note :** indeed, thanks a lot for your vigilance !

The ethics information requires greater precision. You state that the study was approved by departmental authorities of the French Ministry of Education and that all participants provided informed consent, but the manuscript does not identify an ethics committee or institutional review board, an approval or exemption reference number, or the exact form of consent. Please clarify whether formal ethics committee approval was required under French regulations. If the study was exempt from such review, state the relevant basis for the exemption and identify the authority that granted it. Please also specify whether informed consent was obtained electronically, in writing, or by another documented procedure.

**Author’s note :** the ethic section was modified et detailed as per requested.

The Data Availability statement indicates that the data are available from the corresponding author upon reasonable request. However, the submission information states that PLOS requires the data underlying the findings to be made fully available unless a justified legal or ethical restriction applies. Please consider depositing the anonymized minimal dataset and relevant analysis material in an appropriate public repository. If public sharing is restricted, clearly state the specific ethical or legal reason and the procedure through which qualified researchers can obtain access.

**Author’s note :** the data was made available in the supplementary data.

The Statistical Analyses section is not sufficiently detailed to reproduce the analyses. You list Chi- square, Spearman, and Kruskal-Wallis tests, but numerous additional comparisons are reported in the Results, including comparisons of PE duration against the mandatory weekly duration and multiple pairwise comparisons between groups. The statistical tests used for these analyses and for the reported post hoc comparisons are not specified. Please identify the exact statistical test used for each main analysis and explain the post hoc procedure applied after significant omnibus tests.

**Author’s note :** the statistical subsection in the Methods section was entirely rewritten.

A further major statistical concern is the large number of comparisons performed across teacher characteristics, personal physical activity, geographical context, socioeconomic variables, school characteristics, professional competence, training, and several subgroup analyses. With a significance threshold of p < 0.05 and no reported correction for multiple testing, the probability of false-positive findings may be substantial. Please apply an appropriate multiple-comparison strategy where relevant and report effect sizes with confidence intervals rather than relying primarily on p-values.

**Author’s note :** a multivariable model was performed in the revised version. We added a table and completed the Results section as required.

The analyses also appear to assess most variables separately, although many of the investigated factors are likely to be strongly related to one another. Age and seniority, for example, are closely associated, while urban environment, school size, priority education status, external contractors, and department may also be interrelated. Consequently, the reported associations cannot establish whether each factor is independently associated with PE duration. Please consider using an appropriate multivariable regression model to identify independent associations while controlling for major confounders.

**Author’s note :** our multivariable model already adjusts each association on other predictors (age / seniority, urban context / school size / school sector / …)

The hierarchical structure of the data should also be addressed. Teachers may be clustered within the same schools and schools are clustered within six departments, meaning that observations may not be statistically independent. Ignoring this structure can underestimate uncertainty and affect significance testing. Please clarify whether more than one teacher could respond from the same school and, if so, consider a multilevel model or another method that accounts for clustering at school and department levels.

**Author’s note :** this point was addressed in the Limitations section.

The procedure used to remove observations is insufficiently described. You state that responses were retained “after a clean of outliers,” including input errors and duplicates, but you do not report how many observations were removed, which criteria defined an error or an outlier, or how duplicate responses were detected in an entirely anonymous survey. Please provide a transparent participant flow, including the initial number of submitted questionnaires, exclusions by reason, the method used to identify duplicates, and the final analytical sample.

**Author’s note :** this was detailed at the beginning of the Results section.

In the Results section, many statistically significant associations are reported only through p- values, without the corresponding PE durations, group differences, effect sizes, or confidence intervals. Statistical significance alone does not allow the reader to evaluate the magnitude or practical importance of an association. Please report the relevant descriptive statistics for each major comparison together with an appropriate effect size and confidence interval.

**Author’s note :** Methods and Results sections were completed, and a table was added (table 5).

Several potentially important results are described as “data not shown,” including peri-PE duration and some subgroup findings. Because these findings are subsequently interpreted in the Discussion, the reader should be able to examine the underlying results. Please present these analyses in the main manuscript or supplementary material, particularly when they support statements included in the Discussion or Conclusion.

**Author’s note :** these points were addressed in the previous version, and files were added in the supplementary data (numeric data, questionnaire)

There is an important inconsistency regarding external contractors. In the Results, you report that PE duration was longer when an external contractor was present, both in elementary school and preschool. Later in the Discussion, you state that the presence of external contractors “does not augment PE duration.” These statements are contradictory and may substantially alter the interpretation of this factor. Please verify the analysis and revise the Discussion so that it accurately reflects the reported result.

**Author’s note :** these issues were addressed in the discussion.

In the Discussion, several associations are interpreted in causal terms even though the study is observational, cross-sectional in nature, and based on self-reported data. Expressions suggesting that age, seniority, personal physical activity, training, urban context, or school characteristics “impact,” “increase,” or “lower” PE duration imply causality that cannot be demonstrated with this design. Please consistently describe these findings as associations and reserve causal explanations for hypotheses that could be tested in future longitudinal or experimental studies.

**Author’s note :** these issues were corrected throughout the discussion.

Some explanations in the Discussion are also presented with more certainty than the data support. For example, greater experience is assumed to produce greater teaching efficiency, rural environments are assumed to provide more opportunities for outdoor PE, and the presence of USEP is interpreted through possible transfer of skills and affinity. These are plausible interpretations, but they were not directly assessed by your questionnaire. Please clearly identify such explanations as hypotheses and distinguish them from conclusions directly supported by the collected data.

**Author’s note :** modifications were made throughout the discussion as requested.

The interpretation of weather as an obstacle also requires clarification. Teachers frequently reported adverse weather as a barrier, but you later state that national weather data did not appear to explain departmental differences, while the relevant analysis is not presented and is not described in the Methods. Please either provide the weather variables, analytical procedure, and results or remove this additional analysis from the manuscript.

**Author’s note :** we amended the weather section in the discussion.

In the Conclusion, the manuscript again uses wording suggesting that several teacher and school characteristics “impact” PE teaching duration. Given the observational design, low participation proportion, self-reported outcomes, and geographical restriction to six departments, these findings should be presented as associations rather than determinants. Please narrow the conclusion to what your data directly demonstrate and emphasize the exploratory nature of the identified factors.

The final public health statements extend beyond the variables investigated in this study, particularly the suggestion that primary schools are the most relevant institutions for identifying children at risk and acting accordingly. Your study did not assess children’s health status, obesity, cardiovascular risk, or the effectiveness of school-based health screening. Please limit the final interpretation to PE provision, teaching practices, and barriers, and present broader public health implications as potential areas for future research rather than direct conclusions from the present dataset.

**Author’s note :** we modified the conclusion accordingly.

Throughout the manuscript, frequent grammatical, lexical, and syntactic problems occasionally interfere with scientific clarity. Please consider a careful revision by a fluent academic English speaker after the scientific and methodological issues have been addressed, with particular attention to terminology, verb agreement, statistical wording, and consistent use of terms such as “primary school teacher,” “PE duration,” and “training.”

**Author’s note :** we revised the entire manuscript and did our best to fulfil your request.

Thank you for the considerable effort you have invested in this study, and I congratulate you on addressing an important issue concerning Physical Education in primary schools. If you carefully address the methodological, statistical, reporting, and interpretation issues identified above, you will substantially improve the quality, clarity, and scientific credibility of your article. I wish you every success with the revision and with the further development of your work.

**Author’s note :** we sincerely thank the reviewer for the effort and time put in these comments.
