# Supplementary material for: Duration and barriers of physical education teaching in primary schools in 6 French departments
Source: PLoS One. 2026 Nov 3;21(11):e0362195. doi: 10.1371/journal.pone.0362195 (PMC13730362; doi:10.1371/journal.pone.0362195)
Supplement: Supplementary file 4 [file pone.0362195.s004.docx]

**Ref # PONE-D-26-17765**

**“Duration and barriers of physical education teaching in French primary schools”**

**_________________________________________________________________________________**

**Editorial comment :**

- Title. The title does not yet reﬂect the geographical scope of the study as requested by Reviewer #3. Please revise it to explicitly mention that the survey was conducted in six French departments.

**Author’s note :** the title was changed following another reviewer’s request. We changed the title accordingly.

- Causal wording. Several sentences in the Discussion still use causal language (e.g., “impacts”, “augments”, “enhances”, “potent factor”). Given the cross-sectional design, these should be revised to strictly associative wording.

**Author’s note :** the manuscript was modified accordingly.

- English proofreading. Some expressions in the Discussion remain linguistically unnatural and require ﬁnal polishing.

**Author’s note :** the manuscript was entirely revised accordingly (the discussion as requested, but also the introduction).
