# Supplementary material for: Duration and barriers of physical education teaching in primary schools in 6 French departments
Source: PLoS One. 2026 Nov 3;21(11):e0362195. doi: 10.1371/journal.pone.0362195 (PMC13730362; doi:10.1371/journal.pone.0362195)
Supplement: Peer review history

# Peer review: Decision Letter 0

**DOI:** 10.1371/journal.pone.0362195.r001

**Authors:** Francesca D'Elia

4 Jun 2026

Dear Dr. Hermand,

Thank you for submitting your manuscript to PLOS ONE. After careful consideration, we feel that it has merit but does not fully meet PLOS ONE’s publication criteria as it currently stands. Therefore, we invite you to submit a revised version of the manuscript that addresses the points raised during the review process.

Please submit your revised manuscript by Jul 19 2026 11:59PM. If you will need more time than this to complete your revisions, please reply to this message or contact the journal office at plosone@plos.org. When you're ready to submit your revision, log on to https://www.editorialmanager.com/pone/ and select the 'Submissions Needing Revision' folder to locate your manuscript file.

- A letter that responds to each point raised by the academic editor and reviewer(s). You should upload this letter as a separate file labeled 'Response to Reviewers'.
- A marked-up copy of your manuscript that highlights changes made to the original version. You should upload this as a separate file labeled 'Revised Manuscript with Track Changes'.
- An unmarked version of your revised paper without tracked changes. You should upload this as a separate file labeled 'Manuscript'.

If you would like to make changes to your financial disclosure, please include your updated statement in your cover letter. Guidelines for resubmitting your figure files are available below the reviewer comments at the end of this letter.

If applicable, we recommend that you deposit your laboratory protocols in protocols.io to enhance the reproducibility of your results. Protocols.io assigns your protocol its own identifier (DOI) so that it can be cited independently in the future. For instructions see: https://journals.plos.org/plosone/s/submission-guidelines#loc-laboratory-protocols. Additionally, PLOS ONE offers an option for publishing peer-reviewed Lab Protocol articles, which describe protocols hosted on protocols.io. Read more information on sharing protocols at https://plos.org/protocols?utm_medium=editorial-email&utm_source=authorletters&utm_campaign=protocols.

As the corresponding author, your ORCID iD is verified in the submission system and will appear in the published article. PLOS supports the use of ORCID, and we encourage all coauthors to register for an ORCID iD and use it as well. Please encourage your coauthors to verify their ORCID iD within the submission system before final acceptance, as unverified ORCID iDs will not appear in the published article. *Only*  the individual author can complete the verification step; PLOS staff *cannot*  verify ORCID iDs on behalf of authors.

We look forward to receiving your revised manuscript.

Kind regards,

Francesca D'Elia, Ph.D.

Academic Editor

PLOS One

**Journal Requirements:**

1. When submitting your revision, we need you to address these additional requirements.

Please ensure that your manuscript meets PLOS ONE's style requirements, including those for file naming. The PLOS ONE style templates can be found at

https://journals.plos.org/plosone/s/file?id=wjVg/PLOSOne_formatting_sample_main_body.pdf and

https://journals.plos.org/plosone/s/file?id=ba62/PLOSOne_formatting_sample_title_authors_affiliations.pdf

2. Thank you for submitting to PLOS One. We note that you describe receiving explicit, informed consent from participants. Before we can proceed, please specify how consent was witnessed or documented in the questionnaire presentation. For example, was consent written, verbal, did participants have to answer a specific question in the questionnaire granting consent?

3. In the online submission form, you indicated that “This study was performed under the supervision and agreement of Academic departmental authorities.

The data that support the findings of this study are available from the corresponding author, Dr. Eric Hermand, upon reasonable request”.

All PLOS journals now require all data underlying the findings described in their manuscript to be freely available to other researchers, either a. In a public repository, b. Within the manuscript itself, or c. Uploaded as supplementary information.

This policy applies to all data except where public deposition would breach compliance with the protocol approved by your research ethics board. If your data cannot be made publicly available for ethical or legal reasons (e.g., public availability would compromise patient privacy), please explain your reasons on resubmission and your exemption request will be escalated for approval.

4. We note that you have included the phrase “data not shown” in your manuscript. Unfortunately, this does not meet our data sharing requirements. PLOS does not permit references to inaccessible data. We require that authors provide all relevant data within the paper, Supporting Information files, or in an acceptable, public repository. Please add a citation to support this phrase or upload the data that corresponds with these findings to a stable repository (such as Figshare or Dryad) and provide and URLs, DOIs, or accession numbers that may be used to access these data. Or, if the data are not a core part of the research being presented in your study, we ask that you remove the phrase that refers to these data.

5. Please include your full ethics statement in the ‘Methods’ section of your manuscript file. In your statement, please include the full name of the IRB or ethics committee who approved or waived your study, as well as whether or not you obtained informed written or verbal consent. If consent was waived for your study, please include this information in your statement as well.

6. If the reviewer comments include a recommendation to cite specific previously published works, please review and evaluate these publications to determine whether they are relevant and should be cited. There is no requirement to cite these works unless the editor has indicated otherwise.

**Additional Editor Comments:**

Dear Authors,

thank you for submitting your manuscript to PLOS ONE.

Three reviewers have now evaluated your work. They agree that the topic is relevant and that the dataset has potential value for understanding reported PE duration and perceived barriers in French primary schools. At the same time, all reviewers identified several important issues that need to be addressed before the manuscript can be considered further.

In light of the reviewers’ assessments and my own evaluation, I am inviting you to submit a Major Revision.

Please revise the manuscript thoroughly, taking into account all reviewer comments. In particular, the revision should:

- strengthen the methodological and statistical reporting

- improve transparency regarding sampling, questionnaire development, data cleaning, and data availability

- address ethical approval and consent more clearly

- provide more complete and accessible presentation of results

- moderate the interpretation of findings in accordance with the study design

- revise the English language and formatting throughout the manuscript

We appreciate your efforts and look forward to receiving the revised manuscript.

Reviewers' comments:

**Comments to the Author**

1. Is the manuscript technically sound, and do the data support the conclusions?

Reviewer #1: Yes

Reviewer #2: Partly

Reviewer #3: Partly

**********

2. Has the statistical analysis been performed appropriately and rigorously?

Reviewer #1: Yes

Reviewer #2: No

Reviewer #3: No

**********

3. Have the authors made all data underlying the findings in their manuscript fully available?

The PLOS Data policy

Reviewer #1: No

Reviewer #2: No

Reviewer #3: No

**********

4. Is the manuscript presented in an intelligible fashion and written in standard English?

Reviewer #1: Yes

Reviewer #2: No

Reviewer #3: Yes

**********

**Reviewer #1:**  The manuscript addresses an important topic and benefits from a large sample and its focus on PE duration and barriers in French primary schools. However, several revisions are needed before publication.

The title should better reflect the main focus on PE duration and related barriers. The abstract should mention the response rate and its implications for representativeness. The statistical analysis should be strengthened by reporting effect sizes, confidence intervals, adjustment for multiple comparisons, and, preferably, multivariable models. The discussion should avoid causal language because the study is cross-sectional and self-reported, and the limitations should more fully address low response rate, selection bias, self-report bias, and lack of objective PE-duration verification. Finally, the manuscript requires English language editing to correct grammatical and typographical errors. Overall, the study has potential, but methodological clarification, stronger statistical reporting, improved transparency, and language revision are needed.

**Reviewer #2:**  Comments to the Author

I recommend major revision.

The manuscript addresses a relevant topic for education, public health, and school organization. The absolute sample size is substantial, with 1,967 responses from primary teachers across six French departments, and the study provides potentially useful descriptive information on reported physical education duration and perceived barriers to PE teaching. However, several methodological, statistical, and reporting issues currently limit the reliability and interpretability of the findings.

Major comments

1. Data availability

The current data availability statement indicates that the data are available from the corresponding author upon reasonable request. This does not meet the expected standard for PLOS ONE unless clear legal or ethical restrictions prevent public sharing. The authors should deposit the minimal anonymized dataset and, where applicable, the analysis code in a public repository. If public sharing is not possible, the authors should clearly explain the specific restriction, who controls access, how access can be requested, and under what criteria data access would be granted.

2. Ethics approval and informed consent

The ethics statement reports approval by departmental authorities of the French Ministry of Education. However, it is unclear whether these authorities acted as an ethics committee or whether the study received a formal ethics waiver. Since the study involved human participants, even if adult teachers and anonymous responses were used, the authors should clarify the applicable ethical framework, the approving body, whether an approval or waiver number exists, and the exact form of informed consent obtained.

3. Sampling strategy and response rate

The sampling procedure needs further clarification. The manuscript states that teachers from 6,198 schools were contacted, but it is unclear how many individual teachers actually received the invitation. The response rate appears very low, around 3.9 percent overall, with important differences across departments. This raises a serious risk of non-response bias, especially because teachers with a greater interest in PE may have been more likely to participate. The authors should provide the true denominator, explain the recruitment procedure in detail, and, if possible, compare the sample with the target population in terms of sex, age, department, school type, and socioeconomic context.

4. Questionnaire development and validation

The manuscript describes the broad domains covered by the survey, but the questionnaire itself is not provided. The authors should include the complete questionnaire as supplementary material, including the exact wording of items, response options, and any branching logic. They should also describe how the questionnaire was developed, whether it was piloted, whether content validity was assessed, and whether any reliability checks were performed.

5. Main outcome based on self-report

The main outcome, weekly PE duration, is based on teacher self-report. The authors should clarify whether respondents reported a typical week, a planned week, an average across the school year, or another timeframe. It is also important to explain how PE was distinguished from APQ and from peri-PE time. Given the possibility of recall bias and social desirability bias, the limitations of this measure should be discussed more explicitly.

6. Data cleaning and exclusions

The Results section states that responses were retained after cleaning outliers, input errors, and duplicates. However, the manuscript does not explain how many responses were excluded, what criteria were used, or how duplicates were identified in an anonymous survey. The authors should provide a transparent flow diagram showing invited participants, responses received, exclusions, and final analytical sample.

7. Statistical analysis

The statistical analysis is insufficiently described. The authors mention Shapiro-Wilk, Chi-square, Spearman, and Kruskal-Wallis tests, but they do not specify which test was used for each analysis, how post hoc comparisons were conducted, or how multiple comparisons were handled. The manuscript reports many p values, but does not provide effect sizes or confidence intervals. The authors should provide a more detailed statistical analysis plan, including treatment of missing data, assumptions, post hoc procedures, and correction for multiple testing where appropriate.

8. Need for multivariable analyses

Many of the variables examined are likely interrelated, including teacher age, seniority, sex, urban context, priority education network, school size, department, APQ implementation, USEP presence, and use of external contractors. The current analysis appears mostly bivariate. This limits the interpretation of the reported associations. The authors should consider multivariable models to identify factors independently associated with PE duration. If the structure of the data allows it, department or school-level clustering should also be considered.

9. Reporting of descriptive statistics

Table 4 reports mean PE durations by department, but it does not include the number of respondents per subgroup, confidence intervals, medians, or interquartile ranges. Since duration variables may not be normally distributed, robust descriptive statistics would be useful. The authors should provide n values for all subgroup analyses and consider presenting medians and interquartile ranges in addition to means and standard deviations.

10. Results reported as “data not shown”

Several important results are interpreted but not shown, including peri-PE duration, some APQ-related analyses, and other subgroup comparisons. Results that support the main claims should be available in the manuscript, supplementary tables, or the public dataset. The authors should avoid relying on “data not shown” for relevant conclusions.

11. Interpretation of associations

The study design is cross-sectional and observational. Therefore, causal language should be avoided. Several sentences use language such as “impacted”, “increases”, or “lowered”, which may imply causality. The authors should use associative language throughout, such as “was associated with”.

12. Discussion and strength of conclusions

Some interpretations in the Discussion are plausible but not fully supported by the analyses presented. For example, explanations related to rural context, external contractors, multigrade classes, and USEP transfer effects require either additional data or more cautious wording. The conclusion should also be moderated because of the low response rate, voluntary participation, and self-reported nature of the data.

13. Figures

Figures 1 and 2 are pie charts, which are not ideal for comparing multiple categories. Bar charts would improve readability. Figure 2 in particular should be presented as a horizontal bar chart with percentages and n values, and preferably stratified by preschool and elementary school if those differences are discussed in the text.

14. Language and formatting

The manuscript requires substantial English editing. Examples include “questionaries”, “this areas”, “personal et professional”, “teachers’age”, “significative”, “during ,”, “teached”, “non-negligeable”, and “in the same manner than”. The authors should also correct typographical and formatting issues, including the misspelling of Kruskal-Wallis and the apparent error in the reference list for reference 23.

Overall assessment

The manuscript has potential, but substantial revision is required before it can be considered technically sound. The authors should improve data availability, clarify ethics and consent, provide the full questionnaire, transparently report data cleaning and response rates, strengthen the statistical analysis, report effect sizes and confidence intervals, moderate causal interpretations, and revise the English and presentation.

**Reviewer #3:**  Dear Authors,

Thank you for the opportunity to review your manuscript.

Abstract: The health rationale is clear, but the causal wording is too strong when it states that physical activity is the primary defence against sedentary lifestyles and chronic disease. Please soften this claim and state that regular physical activity is an important protective factor against health risks linked to sedentary behaviour.

Introduction: The rationale is relevant, but the research gap needs sharper framing. The manuscript moves from child physical activity to PE teaching duration, yet it does not clearly explain why the selected departments can support broader conclusions about France. Please add a short paragraph that defines the gap, the novelty of this survey, and the limits of the geographic scope.

Materials and methods: The sampling strategy is a major weakness. The response rate is below 4%, and participation relied on voluntary answers, which creates a high risk of selection bias. Please explain how non-response bias was assessed or discuss more clearly that teachers interested in PE may be overrepresented.

The ethics statement needs more precision. The manuscript mentions approval by departmental authorities, but it does not give approval numbers or name a formal ethics committee. Please clarify whether formal ethics review was required, waived, or unavailable, and add the exact approval details where possible.

The questionnaire is not described in enough detail. The reader cannot assess item wording, validation, piloting, or reliability. Please add the questionnaire as supplementary material and describe how it was developed, tested, and distributed.

Statistical analyses: The analysis reports many separate tests across many variables, but it does not mention correction for multiple comparisons or a multivariable model. This raises the risk of false-positive findings. Please consider adding a multivariable regression model and clarify whether p-values were adjusted.

Results: Some results are difficult to verify because several data are mentioned as “not shown.” This weakens transparency, especially for peri-PE duration and pooled obstacles. Please include these data in a table or supplementary file so readers can evaluate the full evidence.

Table and figure presentation needs improvement. Figure 1 appears visually cropped, and Figure 2 is hard to read because part of the pie chart and labels are cut off. Please replace both figures with complete, high-resolution versions and consider bar charts instead of pie charts for clearer comparison.

Discussion: The discussion often interprets associations as if they explain causes. For example, the manuscript suggests reasons for differences by sex, urban context, school size, and external contractors, but the cross-sectional design cannot confirm these mechanisms. Please rephrase causal explanations as hypotheses and keep conclusions aligned with the study design.

Several important confounders remain insufficiently controlled. Department, school size, urban context, priority education status, teacher profile, external contractor presence, and PE affinity may overlap. Please use an adjusted analysis to identify which factors remain associated with PE duration after controlling for the others.

Conclusion: The conclusion states that PE duration is mainly impacted by several factors, but the evidence is observational and self-reported. Please use more cautious wording, such as “was associated with,” and emphasize the descriptive nature of the study.

Data availability: The statement says that data are available from the corresponding author upon reasonable request, but PLOS usually expects the minimal dataset to be publicly available unless legal or ethical restrictions apply. Please deposit the anonymized dataset and code in a public repository, or clearly justify any restriction.

Language and style: The manuscript contains several grammar and wording problems, such as “questionaries,” “appetence,” “significative,” “teached,” “cursus,” and “teachers’age.” Please revise the manuscript with a fluent scientific English editor to improve clarity and consistency.

The main problems concern representativeness, transparency of the questionnaire and data, statistical control of multiple associations, and overinterpretation of cross-sectional findings. Please strengthen these points before the manuscript can support its claims with enough confidence.

**********

what does this mean?). If published, this will include your full peer review and any attached files.

If you choose “no”, your identity will remain anonymous but your review may still be made public.

**Do you want your identity to be public for this peer review?**  For information about this choice, including consent withdrawal, please see our Privacy Policy

Reviewer #1: No

Reviewer #2: No

Reviewer #3: No

**********

[NOTE: If reviewer comments were submitted as an attachment file, they will be attached to this email and accessible via the submission site. Please log into your account, locate the manuscript record, and check for the action link "View Attachments". If this link does not appear, there are no attachment files.]

To ensure your figures meet our technical requirements, please review our figure guidelines: https://journals.plos.org/plosone/s/figures

You may also use PLOS’s free figure tool, NAAS, to help you prepare publication quality figures: https://journals.plos.org/plosone/s/figures#loc-tools-for-figure-preparation.

NAAS will assess whether your figures meet our technical requirements by comparing each figure against our figure specifications.

Attachment Submitted filename: PONE-D-26-17765.pdf

# Author comment: Author response to Decision Letter 1

**DOI:** 10.1371/journal.pone.0362195.r002

17 Jul 2026

Editorial comment :

1. Please ensure that your manuscript meets PLOS ONE's style requirements, including those for file naming. The PLOS ONE style templates can be found at

https://journals.plos.org/plosone/s/file?id=wjVg/PLOSOne_formatting_sample_main_body.pdf and

https://journals.plos.org/plosone/s/file?id=ba62/PLOSOne_formatting_sample_title_authors_affiliations.pdf .

Author’s note : yes, we took the PLOS One formatting into account.

2. Thank you for submitting to PLOS One. We note that you describe receiving explicit, informed consent from participants. Before we can proceed, please specify how consent was witnessed or documented in the questionnaire presentation. For example, was consent written, verbal, did participants have to answer a specific question in the questionnaire granting consent?

Author’s note : we clarified this point in the methodology section.

3. In the online submission form, you indicated that “This study was performed under the supervision and agreement of Academic departmental authorities. The data that support the findings of this study are available from the corresponding author, Dr. Eric Hermand, upon reasonable request”.

All PLOS journals now require all data underlying the findings described in their manuscript to be freely available to other researchers, either a. In a public repository, b. Within the manuscript itself, or c. Uploaded as supplementary information. This policy applies to all data except where public deposition would breach compliance with the protocol approved by your research ethics board. If your data cannot be made publicly available for ethical or legal reasons (e.g., public availability would compromise patient privacy), please explain your reasons on resubmission and your exemption request will be escalated for approval.

Author’s note : after discussions with concerned parties, I will uploaded the necessary data for analyses and interpretation, as a supplementary information.

4. We note that you have included the phrase “data not shown” in your manuscript. Unfortunately, this does not meet our data sharing requirements. PLOS does not permit references to inaccessible data. We require that authors provide all relevant data within the paper, Supporting Information files, or in an acceptable, public repository. Please add a citation to support this phrase or upload the data that corresponds with these findings to a stable repository (such as Figshare or Dryad) and provide and URLs, DOIs, or accession numbers that may be used to access these data. Or, if the data are not a core part of the research being presented in your study, we ask that you remove the phrase that refers to these data.

Author’s note : in addition to data availability in supplementary information and modification of statistical methodology (as required by reviewers), we only kept necessary data to our analyses and further removed unnecessary ones.

5. Please include your full ethics statement in the ‘Methods’ section of your manuscript file. In your statement, please include the full name of the IRB or ethics committee who approved or waived your study, as well as whether or not you obtained informed written or verbal consent. If consent was waived for your study, please include this information in your statement as well.

Author’s note : we clarified this point : as this questionnaire was completely anonymous, did not provide any information allowing to identify any participant, and did not require participants to disclose any racial, ethnic, politic, religious, sexual, health, biometric and genetic information, an ethics committee approval was NOT required.

6. If the reviewer comments include a recommendation to cite specific previously published works, please review and evaluate these publications to determine whether they are relevant and should be cited. There is no requirement to cite these works unless the editor has indicated otherwise.

Author’s note : the reviewers provided useful suggestions and did not require to cite any specific articles. We greatly appreciate that.

Reviewer #1 comments

The manuscript addresses an important topic and benefits from a large sample and its focus on PE duration and barriers in French primary schools. However, several revisions are needed before publication.

1. The title should better reflect the main focus on PE duration and related barriers.

Author’s note : thanks for this suggestion, we modified the title accordingly.

2. The abstract should mention the response rate and its implications for representativeness.

Author’s note : we added this information in the abstract.

3. The statistical analysis should be strengthened by reporting effect sizes, confidence intervals, adjustment for multiple comparisons, and, preferably, multivariable models.

Author’s note : thank you for this suggestion, which is in line with other reviewers’ ones. We completely redesigned the statistical analyses, by including a multivariable regressive model. This allowed us to highlight the best predictors impacting PE duration.

4. The discussion should avoid causal language because the study is cross-sectional and self-reported, and the limitations should more fully address low response rate, selection bias, self-report bias, and lack of objective PE-duration verification.

Author’s note : indeed. We added these limitations at the end of the discussion.

5. Finally, the manuscript requires English language editing to correct grammatical and typographical errors.

Author’s note : this issue was addressed. We also thank reviewers for syntax and grammar suggestions.

Reviewer #2 comments

The manuscript addresses a relevant topic for education, public health, and school organization. The absolute sample size is substantial, with 1,967 responses from primary teachers across six French departments, and the study provides potentially useful descriptive information on reported physical education duration and perceived barriers to PE teaching. However, several methodological, statistical, and reporting issues currently limit the reliability and interpretability of the findings.

1. Data availability. The current data availability statement indicates that the data are available from the corresponding author upon reasonable request. This does not meet the expected standard for PLOS ONE unless clear legal or ethical restrictions prevent public sharing. The authors should deposit the minimal anonymized dataset and, where applicable, the analysis code in a public repository. If public sharing is not possible, the authors should clearly explain the specific restriction, who controls access, how access can be requested, and under what criteria data access would be granted.

Author’s note : after discussion with collaborators, we will provide the data.

2. Ethics approval and informed consent. The ethics statement reports approval by departmental authorities of the French Ministry of Education. However, it is unclear whether these authorities acted as an ethics committee or whether the study received a formal ethics waiver. Since the study involved human participants, even if adult teachers and anonymous responses were used, the authors should clarify the applicable ethical framework, the approving body, whether an approval or waiver number exists, and the exact form of informed consent obtained.

Author’s note : indeed, we were not clear enough about the distinction between the approval and the ethics approvals. Briefly, we needed the approval from departmental authorities to pass the questionnaire on, but no ethics approval was needed because the questionnaire was strictly anonymous and did not ask any questions about racial, ethnic, politic, religious, sexual, health, biometric and genetic background,

3. Sampling strategy and response rate. The sampling procedure needs further clarification. The manuscript states that teachers from 6,198 schools were contacted, but it is unclear how many individual teachers actually received the invitation. The response rate appears very low, around 3.9 percent overall, with important differences across departments. This raises a serious risk of non-response bias, especially because teachers with a greater interest in PE may have been more likely to participate. The authors should provide the true denominator, explain the recruitment procedure in detail, and, if possible, compare the sample with the target population in terms of sex, age, department, school type, and socioeconomic context.

Author’s note : we added a ‘Limitations’ about these matters and detailed some aspects in the methodology section.

4. Questionnaire development and validation. The manuscript describes the broad domains covered by the survey, but the questionnaire itself is not provided. The authors should include the complete questionnaire as supplementary material, including the exact wording of items, response options, and any branching logic. They should also describe how the questionnaire was developed, whether it was piloted, whether content validity was assessed, and whether any reliability checks were performed.

Author’s note : we will provide a questionnaire copy as supplementary material (in French).

5. Main outcome based on self-report. The main outcome, weekly PE duration, is based on teacher self-report. The authors should clarify whether respondents reported a typical week, a planned week, an average across the school year, or another timeframe. It is also important to explain how PE was distinguished from APQ and from peri-PE time. Given the possibility of recall bias and social desirability bias, the limitations of this measure should be discussed more explicitly.

Author’s note : thanks for these remarks, the were addressed in the methodology section and in the limitations section.

6. Data cleaning and exclusions. The Results section states that responses were retained after cleaning outliers, input errors, and duplicates. However, the manuscript does not explain how many responses were excluded, what criteria were used, or how duplicates were identified in an anonymous survey. The authors should provide a transparent flow diagram showing invited participants, responses received, exclusions, and final analytical sample.

Author’s note : these details were clarified in the results and limitations sections.

7. Statistical analysis. The statistical analysis is insufficiently described. The authors mention Shapiro-Wilk, Chi-square, Spearman, and Kruskal-Wallis tests, but they do not specify which test was used for each analysis, how post hoc comparisons were conducted, or how multiple comparisons were handled. The manuscript reports many p values, but does not provide effect sizes or confidence intervals. The authors should provide a more detailed statistical analysis plan, including treatment of missing data, assumptions, post hoc procedures, and correction for multiple testing where appropriate.

Need for multivariable analyses. Many of the variables examined are likely interrelated, including teacher age, seniority, sex, urban context, priority education network, school size, department, APQ implementation, USEP presence, and use of external contractors. The current analysis appears mostly bivariate. This limits the interpretation of the reported associations. The authors should consider multivariable models to identify factors independently associated with PE duration. If the structure of the data allows it, department or school-level clustering should also be considered.

Author’s note : thank you for these suggestions, which were also requested by other reviewers. We deeply modified the statistical analyses and switched to a multivariable analysis, which tuned (and modified) the results. This clearly enhanced the results analysis (despite the extra work !), thanks a lot for this.

9. Reporting of descriptive statistics. Table 4 reports mean PE durations by department, but it does not include the number of respondents per subgroup, confidence intervals, medians, or interquartile ranges. Since duration variables may not be normally distributed, robust descriptive statistics would be useful. The authors should provide n values for all subgroup analyses and consider presenting medians and interquartile ranges in addition to means and standard deviations.

Author’s note : tables and results section were modified accordingly.

10. Results reported as “data not shown”. Several important results are interpreted but not shown, including peri-PE duration, some APQ-related analyses, and other subgroup comparisons. Results that support the main claims should be available in the manuscript, supplementary tables, or the public dataset. The authors should avoid relying on “data not shown” for relevant conclusions.

Author’s note : indeed. These issues were addressed.

11. Interpretation of associations. The study design is cross-sectional and observational. Therefore, causal language should be avoided. Several sentences use language such as “impacted”, “increases”, or “lowered”, which may imply causality. The authors should use associative language throughout, such as “was associated with”.

Author’s note : thank you for the suggestion, we amended our manuscript accordingly.

12. Discussion and strength of conclusions. Some interpretations in the Discussion are plausible but not fully supported by the analyses presented. For example, explanations related to rural context, external contractors, multigrade classes, and USEP transfer effects require either additional data or more cautious wording. The conclusion should also be moderated because of the low response rate, voluntary participation, and self-reported nature of the data.

Author’s note : the modified statistical analysis provided some new interpretations. We used a more cautious wording.

13. Figures. Figures 1 and 2 are pie charts, which are not ideal for comparing multiple categories. Bar charts would improve readability. Figure 2 in particular should be presented as a horizontal bar chart with percentages and n values, and preferably stratified by preschool and elementary school if those differences are discussed in the text.

Author’s note : we first tried bar charts, but they were not very easy to read. Pie charts were finally the best solution for these figures, illustrated with percentages. The multivariable analysis did not point out pre- and elementary schools as significant factors, so there was no need for stratifications.

14. Language and formatting. The manuscript requires substantial English editing. Examples include “questionaries”, “this areas”, “personal et professional”, “teachers’age”, “significative”, “during ,”, “teached”, “non-negligeable”, and “in the same manner than”. The authors should also correct typographical and formatting issues, including the misspelling of Kruskal-Wallis and the apparent error in the reference list for reference 23.

Author’s note : these issues were addressed, thank you for pointing them out.

Reviewer #3 comments

The manuscript addresses an important topic and benefits from a large sample and its focus on PE duration and barriers in French primary schools. However, several revisions are needed before publication.

1. Abstract: The health rationale is clear, but the causal wording is too strong when it states that physical activity is the primary defence against sedentary lifestyles and chronic disease. Please soften this claim and state that regular physical activity is an important protective factor against health risks linked to sedentary behaviour.

Author’s note : these sentences were modified, thanks for pointing them out.

2. Introduction: The rationale is relevant, but the research gap needs sharper framing. The manuscript moves from child physical activity to PE teaching duration, yet it does not clearly explain why the selected departments can support broader conclusions about France. Please add a short paragraph that defines the gap, the novelty of this survey, and the limits of the geographic scope.

Author’s note : we clarified this point in the section leading to the aim and objectives of this study.

3. Materials and methods: The sampling strategy is a major weakness. The response rate is below 4%, and participation relied on voluntary answers, which creates a high risk of selection bias. Please explain how non-response bias was assessed or discuss more clearly that teachers interested in PE may be overrepresented.

Author’s note : these points were discussed the added limitation section.

4. The ethics statement needs more precision. The manuscript mentions approval by departmental authorities, but it does not give approval numbers or name a formal ethics committee. Please clarify whether formal ethics review was required, waived, or unavailable, and add the exact approval details where possible.

Author’s note : indeed, this was clarified in the methodology section. As this questionnaire was completely anonymous, did not provide any information allowing to identify any participant, and did not require participants to disclose any racial, ethnic, politic, religious, sexual, health, biometric and genetic information, an ethics committee approval was NOT required.

5. The questionnaire is not described in enough detail. The reader cannot assess item wording, validation, piloting, or reliability. Please add the questionnaire as supplementary material and describe how it was developed, tested, and distributed.

Author’s note : the questionnaire (in French) was added in the supplemental material.

5. Statistical analyses: The analysis reports many separate tests across many variables, but it does not mention correction for multiple comparisons or a multivariable model. This raises the risk of false-positive findings. Please consider adding a multivariable regression model and clarify whether p-values were adjusted.

Author’s note : thank you for these suggestions, which were also requested by other reviewers. We deeply modified the statistical analyses and switched to a multivariable analysis, which tuned (and modified) the results. This clearly enhanced the results analysis (despite the extra work !), thanks a lot for this.

6. Results: Some results are difficult to verify because several data are mentioned as “not shown.” This weakens transparency, especially for peri-PE duration and pooled obstacles. Please include these data in a table or supplementary file so readers can evaluate the full evidence.

Author’s note : these issues were fixed throughout the manuscript.

7. Table and figure presentation needs improvement. Figure 1 appears visually cropped, and Figure 2 is hard to read because part of the pie chart and labels are cut off. Please replace both figures with complete, high-resolution versions and consider bar charts instead of pie charts for clearer comparison.

Author’s note : I uploaded high-resolution figures (6000x4500 pixels, 600 ppp). I guess the PDF conversion executed by the website was not perfect. We first tried bar charts, but they were not very easy to read. Pie charts were finally the best solution for these figures, illustrated with percentages.

8. Discussion: The discussion often interprets associations as if they explain causes. For example, the manuscript suggests reasons for differences by sex, urban context, school size, and external contractors, but the cross-sectional design cannot confirm these mechanisms. Please rephrase causal explanations as hypotheses and keep conclusions aligned with the study design.

Author’s note : the entire discussion was rewritten in the light of the modified statistical analyses (multivariable model)

9. Several important confounders remain insufficiently controlled. Department, school size, urban context, priority education status, teacher profile, external contractor presence, and PE affinity may overlap. Please use an adjusted analysis to identify which factors remain associated with PE duration after controlling for the others.

Author’s note : We completely redesigned the statistical analyses, by including a multivariable regressive model. This allowed us to highlight the best predictors impacting PE duration.

10. Conclusion: The conclusion states that PE duration is mainly impacted by several factors, but the evidence is observational and self-reported. Please use more cautious wording, such as “was associated with,” and emphasize the descriptive nature of the study.

Author’s note : indeed. We rephrased the concerned sentences.

11. Data availability: The statement says that data are available from the corresponding author upon reasonable request, but PLOS usually expects the minimal dataset to be publicly available unless legal or ethical restrictions apply. Please deposit the anonymized dataset and code in a public repository, or clearly justify any restriction.

Author’s note : after discussions with concerned parties, I will uploaded the necessary data for analyses and interpretation, as a supplementary information

12. Language and style: The manuscript contains several grammar and wording problems, such as “questionaries,” “appetence,” “significative,” “teached,” “cursus,” and “teachers’age.” Please revise the manuscript with a fluent scientific English editor to improve clarity and consistency.

Author’s note : English language was corrected throughout the manuscript.

The main problems concern representativeness, transparency of the questionnaire and data, statistical control of multiple associations, and overinterpretation of cross-sectional findings. Please strengthen these points before the manuscript can support its claims with enough confidence.

Attachment Submitted filename: Responses to reviewers - Rebuttal.docx

# Peer review: Decision Letter 1

**DOI:** 10.1371/journal.pone.0362195.r003

**Authors:** Francesca D'Elia

19 Aug 2026

Dear Dr. Hermand,

Thank you for submitting your manuscript to PLOS One. After careful consideration, we feel that it has merit but does not fully meet PLOS One’s publication criteria as it currently stands. Therefore, we invite you to submit a revised version of the manuscript that addresses the points raised during the review process.

Please submit your revised manuscript by Oct 03 2026 11:59PM. If you will need more time than this to complete your revisions, please reply to this message or contact the journal office at plosone@plos.org. When you're ready to submit your revision, log on to https://www.editorialmanager.com/pone/ and select the 'Submissions Needing Revision' folder to locate your manuscript file.

- A letter that responds to each point raised by the academic editor and reviewer(s). You should upload this letter as a separate file labeled 'Response to Reviewers'.
- A marked-up copy of your manuscript that highlights changes made to the original version. You should upload this as a separate file labeled 'Revised Manuscript with Track Changes'.
- An unmarked version of your revised paper without tracked changes. You should upload this as a separate file labeled 'Manuscript'.

If you would like to make changes to your financial disclosure, please include your updated statement in your cover letter. Guidelines for resubmitting your figure files are available below the reviewer comments at the end of this letter.

If applicable, we recommend that you deposit your laboratory protocols in protocols.io to enhance the reproducibility of your results. Protocols.io assigns your protocol its own identifier (DOI) so that it can be cited independently in the future. For instructions see: https://journals.plos.org/plosone/s/submission-guidelines#loc-laboratory-protocols. Additionally, PLOS One offers an option for publishing peer-reviewed Lab Protocol articles, which describe protocols hosted on protocols.io. Read more information on sharing protocols at https://plos.org/protocols?utm_medium=editorial-email&utm_source=authorletters&utm_campaign=protocols.

As the corresponding author, your ORCID iD is verified in the submission system and will appear in the published article. PLOS supports the use of ORCID, and we encourage all coauthors to register for an ORCID iD and use it as well. Please encourage your coauthors to verify their ORCID iD within the submission system before final acceptance, as unverified ORCID iDs will not appear in the published article. *Only*  the individual author can complete the verification step; PLOS staff *cannot*  verify ORCID iDs on behalf of authors.

We look forward to receiving your revised manuscript.

Kind regards,

Francesca D'Elia, Ph.D.

Academic Editor

PLOS One

Journal Requirements:

If the reviewer comments include a recommendation to cite specific previously published works, please review and evaluate these publications to determine whether they are relevant and should be cited. There is no requirement to cite these works unless the editor has indicated otherwise.

Please review your reference list to ensure that it is complete and correct. If you have cited papers that have been retracted, please include the rationale for doing so in the manuscript text, or remove these references and replace them with relevant current references. Any changes to the reference list should be mentioned in the rebuttal letter that accompanies your revised manuscript. If you need to cite a retracted article, indicate the article’s retracted status in the References list and also include a citation and full reference for the retraction notice.

Additional Editor Comments:

Dear Authors,

Thank you for your thorough revision and for addressing many of the concerns raised in the first review round. Reviewer 1 considers the manuscript suitable for publication, while Reviewers 2 and 3 identify several remaining issues that must be resolved before acceptance.

At this stage, further revisions are required. Please address the following points:

- Provide complete reporting of the multivariable model (coefficients, 95% CIs, and model fit), and correct the remaining statistical inconsistencies.

- Clarify the criteria and numbers for each exclusion during data cleaning.

- Specify clearly how informed consent was documented.

- Revise title, abstract, and wording to reflect the geographical scope, self-reported nature of the data, and avoid causal language.

- Expand methodological details on sampling, response rate calculation, and potential selection bias.

- Correct the inconsistency regarding external contractors in Results vs. Discussion.

- Conduct a final English-language proofreading.

Please submit a revised manuscript together with a point-by-point response to all remaining reviewer comments.

Kind regards,

The Academic Editor

[Note: HTML markup is below. Please do not edit.]

Reviewers' comments:

Reviewer's Responses to Questions

**Comments to the Author**

Reviewer #1: All comments have been addressed

Reviewer #2: All comments have been addressed

Reviewer #3: (No Response)

**********

2. Is the manuscript technically sound, and do the data support the conclusions?

Reviewer #1: Yes

Reviewer #2: Yes

Reviewer #3: Yes

**********

3. Has the statistical analysis been performed appropriately and rigorously?

Reviewer #1: Yes

Reviewer #2: No

Reviewer #3: No

**********

4. Have the authors made all data underlying the findings in their manuscript fully available?

The PLOS Data policy

Reviewer #1: Yes

Reviewer #2: Yes

Reviewer #3: No

**********

5. Is the manuscript presented in an intelligible fashion and written in standard English?

Reviewer #1: Yes

Reviewer #2: No

Reviewer #3: Yes

**********

Reviewer #1: Dear Author,

Following the revisions, I believe that the manuscript has become more robust in terms of its scientific structure, clarity, and overall quality. The revisions have significantly improved the quality of the study and adequately addressed the previously identified issues. Therefore, I consider the manuscript suitable for publication in its current form.

Thank you for your efforts, and I wish you success in your future academic studies.

Best regards.

Reviewer #2: The revised manuscript has improved substantially and most of the previous concerns have been adequately addressed. I have only a few remaining points before publication:

- Statistical reporting (pp. 16–20). Please provide a clearer and more complete report of the multivariable model, including the coefficients and 95% CIs for the retained predictors and an overall measure of model fit. There are also some apparent inconsistencies that should be checked: ten predictors are mentioned but only nine appear to be listed, and the reported partial f² value is negative.

- Results (p. 20). Please correct the remaining statistical/wording inconsistencies. For example, “p>0.027” appears inconsistent with the statement that this comparison was significant. Also, terms such as “influencing,” “augments,” “increase,” and “impact” should be replaced by associative wording given the cross-sectional design.

- Data cleaning (p. 17). The manuscript states that 73 responses were excluded because of input errors, duplicates, and aberrant answers. Please briefly specify the number and criteria for each type of exclusion.

- Consent (pp. 14–15). Please clarify the exact mechanism by which informed consent was documented (e.g., explicit agreement/checkbox before accessing the questionnaire). The current wording appears to describe consent through participation/non-opposition rather than clearly stating how consent was recorded.

- Language. A final English-language proofreading is still needed. Several grammatical and wording issues remain throughout the Abstract, Methods, and Results.

Overall, these are relatively minor issues and the revised manuscript is much stronger.

Reviewer #3: I am glad to review your work, and I thank you for the opportunity to read and evaluate your manuscript.

The title currently suggests that the study provides an overview of Physical Education teaching practices in France, while the survey was conducted in only six French departments and the participation rate was very low. This wording may therefore imply a level of national representativeness that the study design does not support. Please consider specifying the geographical scope in the title, for example by referring to “six French departments,” so that the title accurately reflects the population investigated.

In the Abstract, the main findings are clearly presented, but the study is described without mentioning that PE duration and barriers were self-reported by teachers and that the sample represented only a small proportion of the eligible population. These limitations are important for interpreting the results. Please consider briefly identifying the study as a questionnaire-based survey conducted in six departments and avoiding wording that may imply that the reported values represent all French primary schools.

In the Introduction, the rationale for studying PE duration is relevant, but several statements concerning physical inactivity, sedentary behaviour, obesity, and the protective role of physical activity are presented in rather causal or absolute terms. These concepts are related but not interchangeable, and obesity is influenced by multiple biological, behavioural, social, and environmental factors. Please consider revising these statements so that the background reflects the multifactorial nature of obesity and clearly distinguishes physical inactivity from sedentary behaviour.

In the Materials and Methods section, the sampling strategy and the calculation of the response rate require substantial clarification. You state that teachers from 6,198 primary schools were contacted through school principals and educational authorities, while the reported overall participation proportion is 3.9%. It is unclear whether this percentage was calculated using the total number of teachers in these departments, the number of teachers who actually received the invitation, or another denominator. Please provide the exact number of eligible teachers, the number who received the questionnaire, and the number who responded in each department, and distinguish clearly between a participation proportion and a true response rate.

The very low participation proportion also creates an important risk of nonresponse and self-selection bias. Teachers who chose to complete a voluntary questionnaire about PE may have had greater interest in physical activity or PE teaching than teachers who did not participate. This issue limits the generalizability of the findings considerably. Please compare the characteristics of your sample, where possible, with available characteristics of the overall primary teacher population and discuss more explicitly how selection bias may have influenced PE duration, affinity, competence, and physical activity measures.

The data collection period extended from December 2021 to June 2024, which is particularly important because the educational context changed during this interval. For example, the manuscript states that the Daily Physical Activity initiative, APQ, became prescribed by legislation in 2022. Pooling responses obtained before and after such a policy change may introduce a temporal bias into the analysis. Please clarify when responses were collected within each year and consider analysing or adjusting the results according to the period of data collection, particularly for APQ-related outcomes.

The Survey Design section describes the domains covered by the questionnaire but provides no information about questionnaire development, pilot testing, content validity, reliability, or previous validation. Since most of the conclusions depend directly on self-reported questionnaire data, the measurement properties of the instrument are essential for assessing the reliability of the findings. Please provide the complete questionnaire as supplementary material and explain how the items were developed, whether they were pilot-tested, whether previously validated questions were used, and how the response categories were selected.

The description of the participants should also be revised because the manuscript initially refers to them as “PE teachers,” although the study appears to concern generalist primary school teachers who are responsible for teaching PE as part of the curriculum. This terminology may cause confusion, particularly because external PE contractors are analysed separately later in the manuscript. Please use a consistent term such as “primary school teachers” throughout the manuscript and reserve “PE teacher” for professionally specialised PE teachers if such participants were actually included.

The ethics information requires greater precision. You state that the study was approved by departmental authorities of the French Ministry of Education and that all participants provided informed consent, but the manuscript does not identify an ethics committee or institutional review board, an approval or exemption reference number, or the exact form of consent. Please clarify whether formal ethics committee approval was required under French regulations. If the study was exempt from such review, state the relevant basis for the exemption and identify the authority that granted it. Please also specify whether informed consent was obtained electronically, in writing, or by another documented procedure.

The Data Availability statement indicates that the data are available from the corresponding author upon reasonable request. However, the submission information states that PLOS requires the data underlying the findings to be made fully available unless a justified legal or ethical restriction applies. Please consider depositing the anonymized minimal dataset and relevant analysis material in an appropriate public repository. If public sharing is restricted, clearly state the specific ethical or legal reason and the procedure through which qualified researchers can obtain access.

The Statistical Analyses section is not sufficiently detailed to reproduce the analyses. You list Chi-square, Spearman, and Kruskal-Wallis tests, but numerous additional comparisons are reported in the Results, including comparisons of PE duration against the mandatory weekly duration and multiple pairwise comparisons between groups. The statistical tests used for these analyses and for the reported post hoc comparisons are not specified. Please identify the exact statistical test used for each main analysis and explain the post hoc procedure applied after significant omnibus tests.

A further major statistical concern is the large number of comparisons performed across teacher characteristics, personal physical activity, geographical context, socioeconomic variables, school characteristics, professional competence, training, and several subgroup analyses. With a significance threshold of p < 0.05 and no reported correction for multiple testing, the probability of false-positive findings may be substantial. Please apply an appropriate multiple-comparison strategy where relevant and report effect sizes with confidence intervals rather than relying primarily on p-values.

The analyses also appear to assess most variables separately, although many of the investigated factors are likely to be strongly related to one another. Age and seniority, for example, are closely associated, while urban environment, school size, priority education status, external contractors, and department may also be interrelated. Consequently, the reported associations cannot establish whether each factor is independently associated with PE duration. Please consider using an appropriate multivariable regression model to identify independent associations while controlling for major confounders.

The hierarchical structure of the data should also be addressed. Teachers may be clustered within the same schools and schools are clustered within six departments, meaning that observations may not be statistically independent. Ignoring this structure can underestimate uncertainty and affect significance testing. Please clarify whether more than one teacher could respond from the same school and, if so, consider a multilevel model or another method that accounts for clustering at school and department levels.

The procedure used to remove observations is insufficiently described. You state that responses were retained “after a clean of outliers,” including input errors and duplicates, but you do not report how many observations were removed, which criteria defined an error or an outlier, or how duplicate responses were detected in an entirely anonymous survey. Please provide a transparent participant flow, including the initial number of submitted questionnaires, exclusions by reason, the method used to identify duplicates, and the final analytical sample.

In the Results section, many statistically significant associations are reported only through p-values, without the corresponding PE durations, group differences, effect sizes, or confidence intervals. Statistical significance alone does not allow the reader to evaluate the magnitude or practical importance of an association. Please report the relevant descriptive statistics for each major comparison together with an appropriate effect size and confidence interval.

Several potentially important results are described as “data not shown,” including peri-PE duration and some subgroup findings. Because these findings are subsequently interpreted in the Discussion, the reader should be able to examine the underlying results. Please present these analyses in the main manuscript or supplementary material, particularly when they support statements included in the Discussion or Conclusion.

There is an important inconsistency regarding external contractors. In the Results, you report that PE duration was longer when an external contractor was present, both in elementary school and preschool. Later in the Discussion, you state that the presence of external contractors “does not augment PE duration.” These statements are contradictory and may substantially alter the interpretation of this factor. Please verify the analysis and revise the Discussion so that it accurately reflects the reported result.

In the Discussion, several associations are interpreted in causal terms even though the study is observational, cross-sectional in nature, and based on self-reported data. Expressions suggesting that age, seniority, personal physical activity, training, urban context, or school characteristics “impact,” “increase,” or “lower” PE duration imply causality that cannot be demonstrated with this design. Please consistently describe these findings as associations and reserve causal explanations for hypotheses that could be tested in future longitudinal or experimental studies.

Some explanations in the Discussion are also presented with more certainty than the data support. For example, greater experience is assumed to produce greater teaching efficiency, rural environments are assumed to provide more opportunities for outdoor PE, and the presence of USEP is interpreted through possible transfer of skills and affinity. These are plausible interpretations, but they were not directly assessed by your questionnaire. Please clearly identify such explanations as hypotheses and distinguish them from conclusions directly supported by the collected data.

The interpretation of weather as an obstacle also requires clarification. Teachers frequently reported adverse weather as a barrier, but you later state that national weather data did not appear to explain departmental differences, while the relevant analysis is not presented and is not described in the Methods. Please either provide the weather variables, analytical procedure, and results or remove this additional analysis from the manuscript.

In the Conclusion, the manuscript again uses wording suggesting that several teacher and school characteristics “impact” PE teaching duration. Given the observational design, low participation proportion, self-reported outcomes, and geographical restriction to six departments, these findings should be presented as associations rather than determinants. Please narrow the conclusion to what your data directly demonstrate and emphasize the exploratory nature of the identified factors.

The final public health statements extend beyond the variables investigated in this study, particularly the suggestion that primary schools are the most relevant institutions for identifying children at risk and acting accordingly. Your study did not assess children’s health status, obesity, cardiovascular risk, or the effectiveness of school-based health screening. Please limit the final interpretation to PE provision, teaching practices, and barriers, and present broader public health implications as potential areas for future research rather than direct conclusions from the present dataset.

Throughout the manuscript, frequent grammatical, lexical, and syntactic problems occasionally interfere with scientific clarity. Please consider a careful revision by a fluent academic English speaker after the scientific and methodological issues have been addressed, with particular attention to terminology, verb agreement, statistical wording, and consistent use of terms such as “primary school teacher,” “PE duration,” and “training.”

Thank you for the considerable effort you have invested in this study, and I congratulate you on addressing an important issue concerning Physical Education in primary schools. If you carefully address the methodological, statistical, reporting, and interpretation issues identified above, you will substantially improve the quality, clarity, and scientific credibility of your article. I wish you every success with the revision and with the further development of your work.

**********

what does this mean?). If published, this will include your full peer review and any attached files.

If you choose “no”, your identity will remain anonymous but your review may still be made public.

**Do you want your identity to be public for this peer review?**  For information about this choice, including consent withdrawal, please see our Privacy Policy

Reviewer #1: No

Reviewer #2: No

Reviewer #3: **Yes:**  Stefan Alecu

**********

[NOTE: If reviewer comments were submitted as an attachment file, they will be attached to this email and accessible via the submission site. Please log into your account, locate the manuscript record, and check for the action link "View Attachments". If this link does not appear, there are no attachment files.]

To ensure your figures meet our technical requirements, please review our figure guidelines: https://journals.plos.org/plosone/s/figures

You may also use PLOS’s free figure tool, NAAS, to help you prepare publication quality figures: https://journals.plos.org/plosone/s/figures#loc-tools-for-figure-preparation.

NAAS will assess whether your figures meet our technical requirements by comparing each figure against our figure specifications.

# Author comment: Author response to Decision Letter 2

**DOI:** 10.1371/journal.pone.0362195.r004

8 Sep 2026

Ref # PONE-D-26-17765

“Duration and barriers of physical education teaching in French primary schools”

_________________________________________________________________________________

Editorial comment :

- Provide complete reporting of the multivariable model (coeﬃcients, 95% CIs, and model ﬁt), and correct the remaining statistical inconsistencies.

Author’s note : the Methods section was completed and a table was added.

- Clarify the criteria and numbers for each exclusion during data cleaning.

Author’s note : this was clarified in the Results section.

- Specify clearly how informed consent was documented.

Author’s note : the ethic section was modified et detailed as per requested.

- Revise title, abstract, and wording to reﬂect the geographical scope, self-reported nature of the data, and avoid causal language.

Author’s note : Done.

- Expand methodological details on sampling, response rate calculation, and potential selection bias.

Author’s note : the Methods section was modified accordingly.

- Correct the inconsistency regarding external contractors in Results vs. Discussion.

Author’s note : that issue was addressed.

Reviewer #2 comments

The revised manuscript has improved substantially and most of the previous concerns have been adequately addressed. I have only a few remaining points before publication:

- statistical reporting (pp. 16–20). Please provide a clearer and more complete report of the multivariable model, including the coeﬃcients and 95% CIs for the retained predictors and an overall measure of model ﬁt. There are also some apparent inconsistencies that should be checked: ten predictors are mentioned but only nine appear to be listed, and the reported partial f² value is negative.

Author’s note : the Methods section was completed and a table was added. The issue about predictors was addressed.

- Results (p. 20). Please correct the remaining statistical/wording inconsistencies. for example, “p>0.027” appears inconsistent with the statement that this comparison was signiﬁcant. Also, terms such as “inﬂuencing,” “augments,” “increase,” and “impact” should be replaced by associative wording given the cross-sectional design.

Author’s note : these issues in the Results section were adressed accordingly.

- Data cleaning (p. 17). The manuscript states that 73 responses were excluded because of input errors, duplicates, and aberrant answers. Please brieﬂy specify the number and criteria for each type of exclusion.

Author’s note : this was detailed at the beginning of the results section.

- Consent (pp. 14–15). Please clarify the exact mechanism by which informed consent was documented (e.g., explicit agreement/checkbox before accessing the questionnaire). the current wording appears to describe consent through participation/non-opposition rather than clearly stating how consent was recorded.

Author’s note : the ethic section was modified et detailed as per requested.

- Language. A ﬁnal English-language proofreading is still needed. several grammatical and wording issues remain throughout the Abstract, Methods, and Results.

Author’s note : the manuscript was proof-read accordingly.

Overall, these are relatively minor issues and the revised manuscript is much stronger.

Author’s note : we sincerely thank the reviewer for his/her help in that matter.

Reviewer #3 comments

I am glad to review your work, and I thank you for the opportunity to read and evaluate your manuscript.

The title currently suggests that the study provides an overview of Physical Education teaching practices in France, while the survey was conducted in only six French departments and the participation rate was very low. This wording may therefore imply a level of national representativeness that the study design does not support. Please consider specifying the geographical scope in the title, for example by referring to “six French departments,” so that the title accurately reflects the population investigated.

Author’s note : we modified the title following reviewers’ request.

In the Abstract, the main findings are clearly presented, but the study is described without mentioning that PE duration and barriers were self-reported by teachers and that the sample represented only a small proportion of the eligible population. These limitations are important for interpreting the results. Please consider briefly identifying the study as a questionnaire-based survey conducted in six departments and avoiding wording that may imply that the reported values represent all French primary schools.

Author’s note : we modified the abstract accordingly.

In the Introduction, the rationale for studying PE duration is relevant, but several statements concerning physical inactivity, sedentary behaviour, obesity, and the protective role of physical activity are presented in rather causal or absolute terms. These concepts are related but not interchangeable, and obesity is influenced by multiple biological, behavioural, social, and environmental factors. Please consider revising these statements so that the background reflects the multifactorial nature of obesity and clearly distinguishes physical inactivity from sedentary behaviour.

Author’s note : the introduction was modified accordingly, one reference was added.

In the Materials and Methods section, the sampling strategy and the calculation of the response rate require substantial clarification. You state that teachers from 6,198 primary schools were contacted through school principals and educational authorities, while the reported overall participation proportion is 3.9%. It is unclear whether this percentage was calculated using the total number of teachers in these departments, the number of teachers who actually received the invitation, or another denominator. Please provide the exact number of eligible teachers, the number who received the questionnaire, and the number who responded in each department, and distinguish clearly between a participation proportion and a true response rate.

Author’s note : details were added in the Methods, Results and Limitations sections.

The very low participation proportion also creates an important risk of nonresponse and self- selection bias. Teachers who chose to complete a voluntary questionnaire about PE may have had greater interest in physical activity or PE teaching than teachers who did not participate. This issue limits the generalizability of the findings considerably. Please compare the characteristics of your sample, where possible, with available characteristics of the overall primary teacher population and discuss more explicitly how selection bias may have influenced PE duration, affinity, competence, and physical activity measures.

Author’s note : these points were detailed and explained in the Methods section.

The data collection period extended from December 2021 to June 2024, which is particularly important because the educational context changed during this interval. For example, the manuscript states that the Daily Physical Activity initiative, APQ, became prescribed by legislation in 2022. Pooling responses obtained before and after such a policy change may introduce a temporal bias into the analysis. Please clarify when responses were collected within each year and consider analysing or adjusting the results according to the period of data collection, particularly for APQ-related outcomes.

Author’s note : these points were also covered in the discussion.

The Survey Design section describes the domains covered by the questionnaire but provides no information about questionnaire development, pilot testing, content validity, reliability, or previous validation. Since most of the conclusions depend directly on self-reported questionnaire data, the measurement properties of the instrument are essential for assessing the reliability of the findings. Please provide the complete questionnaire as supplementary material and explain how the items were developed, whether they were pilot-tested, whether previously validated questions were used, and how the response categories were selected.

Author’s note : the questionnaire was added in the supplementary data.

The description of the participants should also be revised because the manuscript initially refers to them as “PE teachers,” although the study appears to concern generalist primary school teachers who are responsible for teaching PE as part of the curriculum. This terminology may cause confusion, particularly because external PE contractors are analysed separately later in the manuscript. Please use a consistent term such as “primary school teachers” throughout the manuscript and reserve “PE teacher” for professionally specialised PE teachers if such participants were actually included.

Author’s note : indeed, thanks a lot for your vigilance !

The ethics information requires greater precision. You state that the study was approved by departmental authorities of the French Ministry of Education and that all participants provided informed consent, but the manuscript does not identify an ethics committee or institutional review board, an approval or exemption reference number, or the exact form of consent. Please clarify whether formal ethics committee approval was required under French regulations. If the study was exempt from such review, state the relevant basis for the exemption and identify the authority that granted it. Please also specify whether informed consent was obtained electronically, in writing, or by another documented procedure.

Author’s note : the ethic section was modified et detailed as per requested.

The Data Availability statement indicates that the data are available from the corresponding author upon reasonable request. However, the submission information states that PLOS requires the data underlying the findings to be made fully available unless a justified legal or ethical restriction applies. Please consider depositing the anonymized minimal dataset and relevant analysis material in an appropriate public repository. If public sharing is restricted, clearly state the specific ethical or legal reason and the procedure through which qualified researchers can obtain access.

Author’s note : the data was made available in the supplementary data.

The Statistical Analyses section is not sufficiently detailed to reproduce the analyses. You list Chi- square, Spearman, and Kruskal-Wallis tests, but numerous additional comparisons are reported in the Results, including comparisons of PE duration against the mandatory weekly duration and multiple pairwise comparisons between groups. The statistical tests used for these analyses and for the reported post hoc comparisons are not specified. Please identify the exact statistical test used for each main analysis and explain the post hoc procedure applied after significant omnibus tests.

Author’s note : the statistical subsection in the Methods section was entirely rewritten.

A further major statistical concern is the large number of comparisons performed across teacher characteristics, personal physical activity, geographical context, socioeconomic variables, school characteristics, professional competence, training, and several subgroup analyses. With a significance threshold of p < 0.05 and no reported correction for multiple testing, the probability of false-positive findings may be substantial. Please apply an appropriate multiple-comparison strategy where relevant and report effect sizes with confidence intervals rather than relying primarily on p-values.

Author’s note : a multivariable model was performed in the revised version. We added a table and completed the Results section as required.

The analyses also appear to assess most variables separately, although many of the investigated factors are likely to be strongly related to one another. Age and seniority, for example, are closely associated, while urban environment, school size, priority education status, external contractors, and department may also be interrelated. Consequently, the reported associations cannot establish whether each factor is independently associated with PE duration. Please consider using an appropriate multivariable regression model to identify independent associations while controlling for major confounders.

Author’s note : our multivariable model already adjusts each association on other predictors (age / seniority, urban context / school size / school sector / …)

The hierarchical structure of the data should also be addressed. Teachers may be clustered within the same schools and schools are clustered within six departments, meaning that observations may not be statistically independent. Ignoring this structure can underestimate uncertainty and affect significance testing. Please clarify whether more than one teacher could respond from the same school and, if so, consider a multilevel model or another method that accounts for clustering at school and department levels.

Author’s note : this point was addressed in the Limitations section.

The procedure used to remove observations is insufficiently described. You state that responses were retained “after a clean of outliers,” including input errors and duplicates, but you do not report how many observations were removed, which criteria defined an error or an outlier, or how duplicate responses were detected in an entirely anonymous survey. Please provide a transparent participant flow, including the initial number of submitted questionnaires, exclusions by reason, the method used to identify duplicates, and the final analytical sample.

Author’s note : this was detailed at the beginning of the Results section.

In the Results section, many statistically significant associations are reported only through p- values, without the corresponding PE durations, group differences, effect sizes, or confidence intervals. Statistical significance alone does not allow the reader to evaluate the magnitude or practical importance of an association. Please report the relevant descriptive statistics for each major comparison together with an appropriate effect size and confidence interval.

Author’s note : Methods and Results sections were completed, and a table was added (table 5).

Several potentially important results are described as “data not shown,” including peri-PE duration and some subgroup findings. Because these findings are subsequently interpreted in the Discussion, the reader should be able to examine the underlying results. Please present these analyses in the main manuscript or supplementary material, particularly when they support statements included in the Discussion or Conclusion.

Author’s note : these points were addressed in the previous version, and files were added in the supplementary data (numeric data, questionnaire)

There is an important inconsistency regarding external contractors. In the Results, you report that PE duration was longer when an external contractor was present, both in elementary school and preschool. Later in the Discussion, you state that the presence of external contractors “does not augment PE duration.” These statements are contradictory and may substantially alter the interpretation of this factor. Please verify the analysis and revise the Discussion so that it accurately reflects the reported result.

Author’s note : these issues were addressed in the discussion.

In the Discussion, several associations are interpreted in causal terms even though the study is observational, cross-sectional in nature, and based on self-reported data. Expressions suggesting that age, seniority, personal physical activity, training, urban context, or school characteristics “impact,” “increase,” or “lower” PE duration imply causality that cannot be demonstrated with this design. Please consistently describe these findings as associations and reserve causal explanations for hypotheses that could be tested in future longitudinal or experimental studies.

Author’s note : these issues were corrected throughout the discussion.

Some explanations in the Discussion are also presented with more certainty than the data support. For example, greater experience is assumed to produce greater teaching efficiency, rural environments are assumed to provide more opportunities for outdoor PE, and the presence of USEP is interpreted through possible transfer of skills and affinity. These are plausible interpretations, but they were not directly assessed by your questionnaire. Please clearly identify such explanations as hypotheses and distinguish them from conclusions directly supported by the collected data.

Author’s note : modifications were made throughout the discussion as requested.

The interpretation of weather as an obstacle also requires clarification. Teachers frequently reported adverse weather as a barrier, but you later state that national weather data did not appear to explain departmental differences, while the relevant analysis is not presented and is not described in the Methods. Please either provide the weather variables, analytical procedure, and results or remove this additional analysis from the manuscript.

Author’s note : we amended the weather section in the discussion.

In the Conclusion, the manuscript again uses wording suggesting that several teacher and school characteristics “impact” PE teaching duration. Given the observational design, low participation proportion, self-reported outcomes, and geographical restriction to six departments, these findings should be presented as associations rather than determinants. Please narrow the conclusion to what your data directly demonstrate and emphasize the exploratory nature of the identified factors.

The final public health statements extend beyond the variables investigated in this study, particularly the suggestion that primary schools are the most relevant institutions for identifying children at risk and acting accordingly. Your study did not assess children’s health status, obesity, cardiovascular risk, or the effectiveness of school-based health screening. Please limit the final interpretation to PE provision, teaching practices, and barriers, and present broader public health implications as potential areas for future research rather than direct conclusions from the present dataset.

Author’s note : we modified the conclusion accordingly.

Throughout the manuscript, frequent grammatical, lexical, and syntactic problems occasionally interfere with scientific clarity. Please consider a careful revision by a fluent academic English speaker after the scientific and methodological issues have been addressed, with particular attention to terminology, verb agreement, statistical wording, and consistent use of terms such as “primary school teacher,” “PE duration,” and “training.”

Author’s note : we revised the entire manuscript and did our best to fulfil your request.

Thank you for the considerable effort you have invested in this study, and I congratulate you on addressing an important issue concerning Physical Education in primary schools. If you carefully address the methodological, statistical, reporting, and interpretation issues identified above, you will substantially improve the quality, clarity, and scientific credibility of your article. I wish you every success with the revision and with the further development of your work.

Author’s note : we sincerely thank the reviewer for the effort and time put in these comments.

Attachment Submitted filename: Responses to reviewers - Rebuttal2.docx

# Editor report: Decision Letter 2

**DOI:** 10.1371/journal.pone.0362195.r005

**Authors:** Francesca D'Elia

14 Oct 2026

Dear Dr. Hermand,

Thank you for submitting your manuscript to PLOS One. After careful consideration, we feel that it has merit but does not fully meet PLOS One’s publication criteria as it currently stands. Therefore, we invite you to submit a revised version of the manuscript that addresses the points raised during the review process.

Please submit your revised manuscript by Nov 28 2026 11:59PM. If you will need more time than this to complete your revisions, please reply to this message or contact the journal office at plosone@plos.org. When you're ready to submit your revision, log on to https://www.editorialmanager.com/pone/ and select the 'Submissions Needing Revision' folder to locate your manuscript file.

- A letter that responds to each point raised by the academic editor and reviewer(s). You should upload this letter as a separate file labeled 'Response to Reviewers'.
- A marked-up copy of your manuscript that highlights changes made to the original version. You should upload this as a separate file labeled 'Revised Manuscript with Track Changes'.
- An unmarked version of your revised paper without tracked changes. You should upload this as a separate file labeled 'Manuscript'.

If you would like to make changes to your financial disclosure, please include your updated statement in your cover letter. Guidelines for resubmitting your figure files are available below the reviewer comments at the end of this letter.

If applicable, we recommend that you deposit your laboratory protocols in protocols.io to enhance the reproducibility of your results. Protocols.io assigns your protocol its own identifier (DOI) so that it can be cited independently in the future. For instructions see: https://journals.plos.org/plosone/s/submission-guidelines#loc-laboratory-protocols. Additionally, PLOS One offers an option for publishing peer-reviewed Lab Protocol articles, which describe protocols hosted on protocols.io. Read more information on sharing protocols at https://plos.org/protocols?utm_medium=editorial-email&utm_source=authorletters&utm_campaign=protocols.

As the corresponding author, your ORCID iD is verified in the submission system and will appear in the published article. PLOS supports the use of ORCID, and we encourage all coauthors to register for an ORCID iD and use it as well. Please encourage your coauthors to verify their ORCID iD within the submission system before final acceptance, as unverified ORCID iDs will not appear in the published article. *Only*  the individual author can complete the verification step; PLOS staff *cannot*  verify ORCID iDs on behalf of authors.

We look forward to receiving your revised manuscript.

Kind regards,

Francesca D'Elia, Ph.D.

Academic Editor

PLOS One

Journal Requirements:

If the reviewer comments include a recommendation to cite specific previously published works, please review and evaluate these publications to determine whether they are relevant and should be cited. There is no requirement to cite these works unless the editor has indicated otherwise.

Please review your reference list to ensure that it is complete and correct. If you have cited papers that have been retracted, please include the rationale for doing so in the manuscript text, or remove these references and replace them with relevant current references. Any changes to the reference list should be mentioned in the rebuttal letter that accompanies your revised manuscript. If you need to cite a retracted article, indicate the article’s retracted status in the References list and also include a citation and full reference for the retraction notice.

Additional Editor Comments:

Dear Authors, thank you for your thorough revision of the manuscript. The paper has improved substantially and most reviewer comments have been adequately addressed. However, a few minor issues still need to be resolved:

1. Title. The title does not yet reflect the geographical scope of the study as requested by Reviewer #3. Please revise it to explicitly mention that the survey was conducted in six French departments.

2. Causal wording. Several sentences in the Discussion still use causal language (e.g., “impacts”, “augments”, “enhances”, “potent factor”). Given the cross-sectional design, these should be revised to strictly associative wording.

3. English proofreading. Some expressions in the Discussion remain linguistically unnatural and require final polishing.

[Note: HTML markup is below. Please do not edit.]

[NOTE: If reviewer comments were submitted as an attachment file, they will be attached to this email and accessible via the submission site. Please log into your account, locate the manuscript record, and check for the action link "View Attachments". If this link does not appear, there are no attachment files.]

To ensure your figures meet our technical requirements, please review our figure guidelines: https://journals.plos.org/plosone/s/figures

You may also use PLOS’s free figure tool, NAAS, to help you prepare publication quality figures: https://journals.plos.org/plosone/s/figures#loc-tools-for-figure-preparation.

NAAS will assess whether your figures meet our technical requirements by comparing each figure against our figure specifications.

# Author comment: Author response to Decision Letter 3

**DOI:** 10.1371/journal.pone.0362195.r006

15 Oct 2026

Ref # PONE-D-26-17765

“Duration and barriers of physical education teaching in French primary schools”

_________________________________________________________________________________

Editorial comment :

- Title. The title does not yet reﬂect the geographical scope of the study as requested by Reviewer #3. Please revise it to explicitly mention that the survey was conducted in six French departments.

Author’s note : the title was changed following another reviewer’s request. We changed the title accordingly.

- Causal wording. Several sentences in the Discussion still use causal language (e.g., “impacts”, “augments”, “enhances”, “potent factor”). Given the cross-sectional design, these should be revised to strictly associative wording.

Author’s note : the manuscript was modified accordingly.

- English proofreading. Some expressions in the Discussion remain linguistically unnatural and require ﬁnal polishing.

Author’s note : the manuscript was entirely revised accordingly (the discussion as requested, but also the introduction).

Attachment Submitted filename: Responses to reviewers - Rebuttal3.docx

# Editor report: Decision Letter 3

**DOI:** 10.1371/journal.pone.0362195.r007

**Authors:** Francesca D'Elia

22 Oct 2026

Duration and barriers of physical education teaching in primary schools in 6 French departments

PONE-D-26-17765R3

Dear Dr. Hermand,

We’re pleased to inform you that your manuscript has been judged scientifically suitable for publication and will be formally accepted for publication once it meets all outstanding technical requirements.

Within one week, you’ll receive an e-mail detailing the required amendments. When these have been addressed, you’ll receive a formal acceptance letter and your manuscript will be scheduled for publication.

An invoice will be generated when your article is formally accepted. Please note, if your institution has a publishing partnership with PLOS and your article meets the relevant criteria, all or part of your publication costs will be covered. Please make sure your user information is up-to-date by logging into Editorial Manager at Editorial Manager® and clicking the ‘Update My Information' link at the top of the page. For questions related to billing, please contact billing support.

If your institution or institutions have a press office, please notify them about your upcoming paper to help maximize its impact. If they’ll be preparing press materials, please inform our press team as soon as possible -- no later than 48 hours after receiving the formal acceptance. Your manuscript will remain under strict press embargo until 2 pm Eastern Time on the date of publication. For more information, please contact onepress@plos.org.

Kind regards,

Francesca D'Elia, Ph.D.

Academic Editor

PLOS One

Additional Editor Comments (optional):

Dear Authors,

Thank you for your careful revisions and detailed responses throughout the review process. The reviewers' and editorial comments have been satisfactorily addressed, and the manuscript has been substantially improved.

I am pleased to recommend acceptance of the manuscript for publication.

Kind regards

The Academic Editor

Francesca D'Elia

Reviewers' comments:

# Editor report: Acceptance letter

**DOI:** 10.1371/journal.pone.0362195.r008

**Authors:** Francesca D'Elia

PONE-D-26-17765R3

PLOS One

Dear Dr. Hermand,

I'm pleased to inform you that your manuscript has been deemed suitable for publication in PLOS One. Congratulations! Your manuscript is now being handed over to our production team.

At this stage, our production department will prepare your paper for publication. This includes ensuring the following:

* All references, tables, and figures are properly cited

* All relevant supporting information is included in the manuscript submission,

* There are no issues that prevent the paper from being properly typeset

You will receive further instructions from the production team, including instructions on how to review your proof when it is ready. Please keep in mind that we are working through a large volume of accepted articles, so please give us a few days to review your paper and let you know the next and final steps.

Lastly, if your institution or institutions have a press office, please let them know about your upcoming paper now to help maximize its impact. If they'll be preparing press materials, please inform our press team within the next 48 hours. Your manuscript will remain under strict press embargo until 2 pm Eastern Time on the date of publication. For more information, please contact onepress@plos.org.

You will receive an invoice from PLOS for your publication fee after your manuscript has reached the completed accept phase. If you receive an email requesting payment before acceptance or for any other service, this may be a phishing scheme. Learn how to identify phishing emails and protect your accounts at https://explore.plos.org/phishing.

If we can help with anything else, please email us at customercare@plos.org.

Thank you for submitting your work to PLOS One and supporting open access.

Kind regards,

PLOS One Editorial Office Staff

on behalf of

Dr. Francesca D'Elia

Academic Editor

PLOS One
